# *In vivo* safety study using radiation at wavelengths and dosages relevant to intravascular imaging

**DOI:** 10.1117/1.JBO.27.1.016003

**Published:** 2022-01-31

**Authors:** Timothy Sowers, Don VanderLaan, Andrei Karpiouk, Daisuke Onohara, Susan Schmarkey, Serge Rousselle, Muralidhar Padala, Stanislav Emelianov

**Affiliations:** a Georgia Institute of Technology, Parker H. Petit Institute for Bioengineering and Bioscience, Atlanta, Georgia, United States; b Georgia Institute of Technology, George W. Woodruff School of Mechanical Engineering, Atlanta, Georgia, United States; c Georgia Institute of Technology, School of Electrical and Computer Engineering, Atlanta, Georgia, United States; d Emory University Hospital Midtown, Structural Heart Research and Innovation Laboratory, Carlyle Fraser Heart Center, Atlanta, Georgia, United States; e StageBio, Mt. Jackson, Virginia, United States; f Georgia Institute of Technology and Emory University School of Medicine, Wallace H. Coulter Department of Biomedical Engineering, Atlanta, Georgia, United States; g Emory University School of Medicine, Division of Cardiothoracic Surgery, Department of Surgery, Atlanta, Georgia, United States

**Keywords:** imaging, intravascular, photoacoustics, laser, safety, *in vivo*

## Abstract

**Significance:**

Intravascular photoacoustic (IVPA) imaging can identify native lipid in atherosclerotic plaques *in vivo*. However, the large number of laser pulses required to produce 3D images is a safety concern that has not been fully addressed.

**Aim:**

We aim to evaluate if irradiation at wavelengths and dosages relevant to IVPA imaging causes target vessel damage.

**Approach:**

We irradiate the carotid artery of swine at one of several energy dosages using radiation at 1064 or 1720 nm and use histological evaluation by a pathologist to identify dose-dependent damage.

**Results:**

Media necrosis was the only dose-dependent form of injury. Damage was present at a cumulative fluence of $50\;J/{\mathrm{cm}}^{2}$ when using 1720 nm light. Damage was more equivocally identified at $700\;J/{\mathrm{cm}}^{2}$ using 1064 nm.

**Conclusions:**

In prior work, IVPA imaging of native lipid in swine has been successfully conducted below the damage thresholds identified. This indicates that it will be possible to use IVPA imaging in a clinical setting without damaging vessel tissue. Future work should determine if irradiation causes an increase in blood thrombogenicity and confirm whether damaged tissue will heal over longer time points.

## 1 Introduction

Intravascular photoacoustic (IVPA) imaging has been developed over the last decade to characterize lipid in atherosclerotic plaques.^1^^–^^12^ Technical advances in the field have continued in recent work.^13^ This includes improvements to catheter designs that focus light, improve resolution, and incorporate multiple element transducers.^14^^–^^16^ Other researchers are developing systems that combine therapy with identification of plaques from IVPA imaging.^17^ Contrast agents have been tested, which can be used to enhance plaque imaging.^18^ Researchers have also developed systems that combine IVPA imaging with other modalities such as optical coherence tomography or near-infrared fluorescence.^19^^,^^20^

IVPA’s specificity to lipid and ability to spatially register the signal with depth makes it possible to map the 3D shape and volume of lipid in the vessel wall. IVPA imaging has been successfully used to image lipid *in vivo* in swine,^4^^,^^11^ including native lipid in atherosclerotic plaques.^1^ Recent prospective studies have shown that direct measurements of lipid in plaques can be used to improve prediction of future cardiovascular events using alternative lipid specific imaging modalities.^21^ Thus IVPA could be uniquely valuable as a diagnostic tool in the clinic.

However, the dosage of light, defined here as the cumulative fluence over an entire imaging sequence, that can be used for IVPA imaging without causing damage to tissue is unknown. This is a concern because 3D volumetric IVPA imaging can require that a single region of tissue be irradiated hundreds of times while the catheter is rotated and retracted.^8^ Although standards for irradiation of human tissue exist (ANSI Z136.1: Safe Use of Lasers),^22^ the focus is on exposure limits for skin and ocular exposure rather than blood vessel tissue. A demonstration of safety on vessel tissue is a necessary step before translating IVPA imaging to the clinic. The study must use light near the lipid wavelength peaks of 1210 or 1720 nm. It would also be useful to test at 1064 nm since Nd:YAG lasers make this is an easily accessible wavelength for multiple wavelength identification of lipid.^6^^,^^23^^,^^24^

In previous work, we investigated the safety of IVPA for the first time *in vitro* using radiation applied to cell types found in vessel tissue.^25^ The study had two main outcomes. First, it showed that any damage from IVPA would be caused by heat accumulation over many pulses rather than from high optical fluence from single pulses. Second, it established dosage thresholds for light at 1064 and 1197 nm. However, the threshold for tissue damage in IVPA imaging has not been evaluated in a realistic *in vivo* environment. It would be useful for researchers developing IVPA to know the energy dosages and imaging protocols that would be safe.

We describe here a set of experiments designed to determine the dosage threshold for damage to vessel tissue from light radiation in a wavelength and dose-dependent manner. The experiments were conducted on the carotid arteries of swine *in vivo*. The carotid arteries were irradiated at either 1064 or 1720 nm. At each wavelength, 1 of 3 dosages was applied to different regions of tissue. The dosages were chosen based on the damage thresholds found in the cell studies we conducted previously.^25^ The arteries were excised and evaluated by a pathologist. The conclusions from that evaluation and its implications for clinical translation of IVPA imaging are discussed.

## 2 Materials and Methods

### 2.1 Laser System and Catheter

Light radiation was produced with a high-power, high pulse repetition rate (PRF) custom made laser (Ekspla, NT200). The laser consisted of an Nd:YAG pumped optical parametric oscillator capable of producing light in the wavelength range from 1690 to 1750 nm. The light from the Nd:YAG at 1064 nm could also be recovered from a side port in the laser. The laser was operated at a PRF of 3840 Hz for all tests. Light from all wavelengths was optically coupled into the same 300  μm optical fiber, which in turn was optically coupled to the catheter using fiber-to-fiber coupling. The fiber-to-fiber coupling was implemented by fixing, end-facing-end, the polished tips of each fiber with minimal (<200  μm) separation. As the catheter rotates within its sheath, light from the stationary fiber is coupled into the rotating fiber. Upon exiting the catheter, the maximum pulse energy was 0.55 mJ at 1064 nm and 0.22 mJ at 1720 nm. The energy was measured using an energy meter with the incident light having a spot size of <1  mm.

**Fig. 1:**
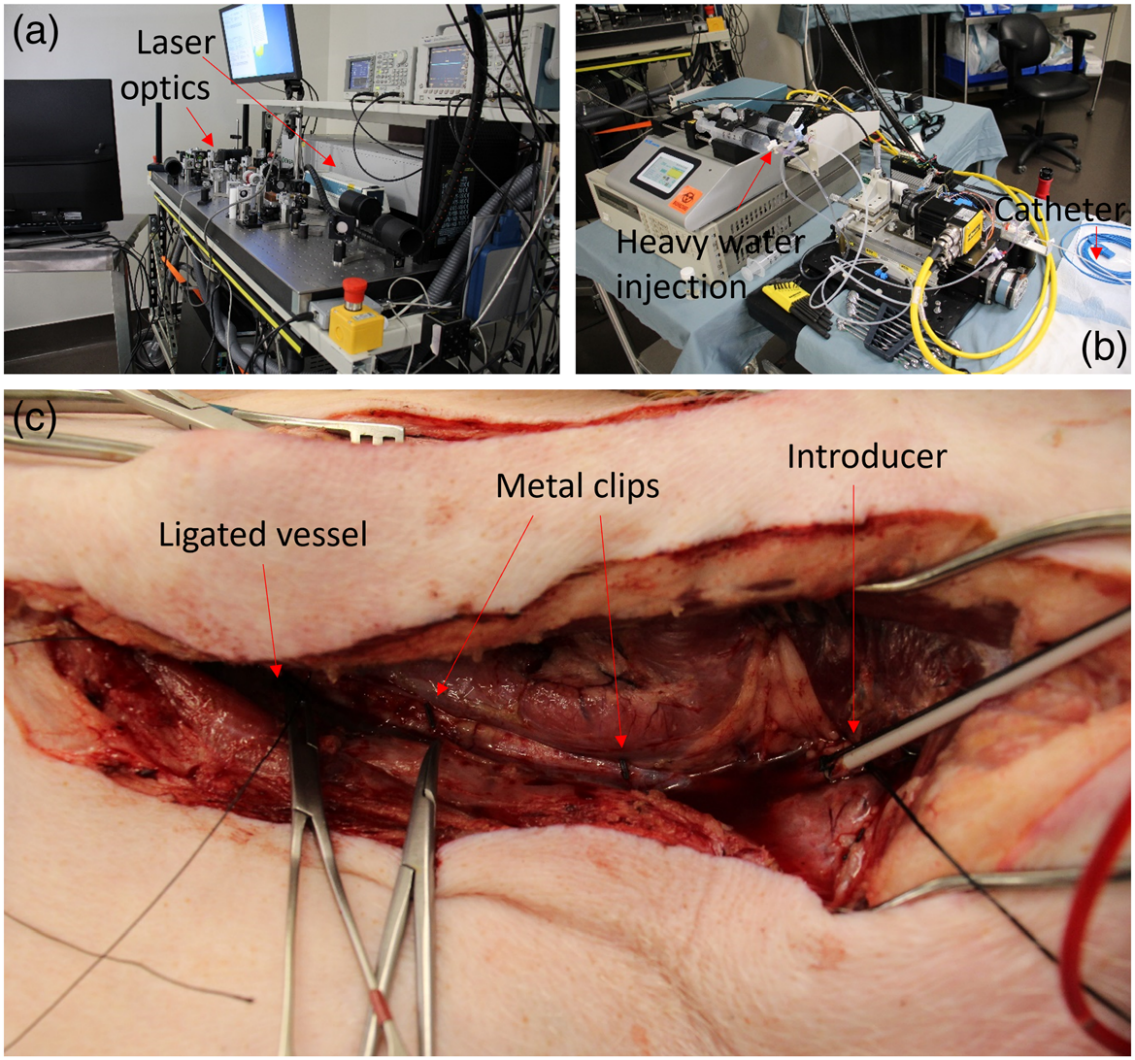
(a) An image of the laser and imaging system used for IVPA imaging, including the laser, optics, and computational hardware. (b) An image of components of the imaging system kept at the side of the operating table. The components include linear and rotational motors, a pumping system for injection of heavy water, and the catheter, which is extending to the bed at the right of the image. (c) Image of one of the carotid arteries after tissue dissection. The vessel has been ligated on the inferior (left) side to isolate the artery after catheter introduction (right). Two metal clips are attached to the artery for registration of fluoroscopy images.

Because of the large size of the laser and optics, the system was split into two parts. The first part [Fig. 1(a)] consisted of the laser, optical components to couple light into the optical fiber, and computational hardware for running the laser and imaging system. The second part [Fig. 1(b)] was small enough to be placed on a cart next to the surgical bed. It included a spindle and linear stage, which were used to rotate and retract the catheter. The spindle electrically and optically connected the catheter to the computer system, electrical hardware, and laser on the nonbedside portion of the system.

Light was delivered to the vessel tissue through a catheter. The catheter consisted of a torque cable (Asahi Intecc USA, Inc.) containing the 300-μm core optical fiber for light delivery. The end of the optical fiber was polished to 37 deg, which resulted in side-firing delivery of light from the catheter due to total internal reflection, as has been described previously.^3^ The tip of the optical fiber was encapsulated in a glass cap held in place by epoxy. The light exiting the catheter was not focused. This optical fiber extended just beyond the end of the torque cable so that emitted light was not obstructed. The torque cable of the catheter was contained in a polyethylene sheath (BD Medical, 63018-781) to prevent damage to vessel tissue during catheter rotation. The proximal end of the polyethylene sheath was constructed to allow rotation of the catheter and to provide a cannula for injection of flushing fluid into the vessel. Fluid injection prevented backflow of blood into the catheter, which could result in blood stasis and coagulation.

### 2.2 Animal Model and Procedure

Two Yorkshire swine were used for the safety study. Anesthesia was induced with 5% isoflurane in 100% oxygen, with the animal intubated, mechanically ventilated, and placed in the supine position on the operating table. Anesthesia was maintained at 1.5% to 2% isoflurane and thereafter supplemented with propofol as needed. In the first swine, only the right carotid artery was irradiated. In the second pig, both carotid arteries were irradiated. The laser radiation was applied to the inside of the carotid arteries of the swine, so a longitudinal neck incision was performed followed by tissue dissection until about 10 cm of the carotid artery was exposed. The catheter was advanced into the vessel through a 7 Fr introducer inserted into the superior side of the exposed vessel. After irradiation, the pigs were euthanized using KCl or Euthasol while under deep anesthesia.

Before irradiation, blood had to be removed from the lumen of the carotid artery. This is because blood has high attenuation at IVPA relevant wavelengths. As a result, much of the light dosage would be absorbed by blood before reaching tissue if blood were present. To prevent this, the inferior end of the carotid artery was ligated [Fig. 1(c)]. With the artery segment isolated, heavy water (deuterium oxide, $D_{2}O$) was injected through the cannula between the torque cable and polyethylene sheath to flush blood from the artery. Heavy water is a nontoxic and nonscattering medium and has a low attenuation coefficient at both 1064 and 1720 nm.^26^ The artery was first cleared of blood with a 50-ml bolus flush of heavy water. Then during the full length of the safety studies, heavy water was continuously flushed into the artery at a rate of 400 ml/h. The blood/water mixture was able to exit the artery through the cannula between the introducer used to insert the catheter into the carotid artery and the polyethylene sheath around the torque cable of the catheter.

Tissues were irradiated with six wavelength and dosage combinations. The wavelengths used were 1064 and 1720 nm. Wavelengths near 1720 nm are used for lipid imaging. Tissue has a higher absorption coefficient at this wavelength than the other lipid imaging wavelength region at 1210 nm, so more heat will be generated in the tissue for a given radiation dosage.^27^ This makes 1720 nm the more conservative wavelength to use for the safety study since a safe dosage at 1720 nm should be safe at 1210 nm. The 1064-nm wavelength was chosen because it is commonly available with the Nd:YAG laser, thus making it desirable for dual wavelength imaging. Three dosages were chosen for each wavelength (Table 1), which were calculated using the pulse energy measured out of the fiber. These dosages were chosen based on cell studies conducted previously.^25^ The dosages were chosen such that the highest would be expected to cause damage, whereas the lowest would be well below the expected damage threshold. The wide range of dosages chosen were due to the large variation in dosages that can occur when choosing different imaging parameters (e.g., A-lines per frame, pullback rate, and energy per pulse), as we discussed in our previous work on cells.^25^ The maximum dosage at 1064 nm in this study is larger because a higher dosage was required to damage cells in that study, presumably due to the higher absorption coefficient at 1720 nm. Each segment of tissue was irradiated with only one wavelength and at one dosage as the catheter was rotated and retracted during irradiation.

**Table 1:** Wavelength and light dosage conditions with sample sizes for each condition. There are a total of 48 samples, 16 from each of the 3 carotid arteries that were irradiated.

Condition number	Wavelength (nm)	Fluence ($J/{\mathrm{cm}}^{2}$)	Sample number
C0	None	0	9
C1	1064	8.3	9
C2		100	7
C3		700	5
C4	1720	8.3	4
C5		50	10
C6		200	4

Fluoroscopy was used to track the location along the artery where each dosage was applied. Metal clips were placed on the viscera of the artery 5 to 6 cm apart to act as reference points in the fluoroscopy image [Fig. 1(c)]. This was particularly important, because the spring-like nature of the torque cable made it so that the distance the catheter was retracted mechanically at the bedside system did not always match the distance the catheter tip retracted in the body. Two metal clips were attached to adventitial tissue at each end of the carotid artery, separated at a known distance. Fluoroscopy images were taken before and after each dosage was applied to the vessel wall. Since the clips in the fluoroscopy image were separated by a known distance, it was possible to convert distance in the fluoroscopy image (in pixels) to a physical length (mm). This information was used to identify the location where each dosage applied to a segment of tissue after the artery was excised. The clips also acted as reference points to measure the length of the artery before and after excision, which made it possible to account for vessel shrinkage. One additional side effect of the spring-like behavior of the torque cable was that not all conditions had the same sample size. The sample size for each condition is listed in Table 1.

All surgeries were conducted in accordance with the IACUC protocol EM78P at the Structural Heart Research Laboratories at Emory University. Approval for off-campus animal work was given by the IACUC at the Georgia Institute of Technology.

### 2.3 Tissue Preparation and Pathology

The carotid arteries were removed after euthanasia and fixed in 10% neutral buffered formalin. The two carotid arteries irradiated in the second pig were stretched and pinned to a board of cork at their original length, determined using the metal clips. The carotid artery in the first pig was assumed to have undergone uniform shrinkage, and the pullback distances in the fluoroscopy images were adjusted accordingly.

Each of the 3 vessels was cut into 16 segments, and the fluoroscopy images were used to determine which dosage was applied to each segment. The segments were labeled such that the pathologist was blinded to the dosage applied to each segment. The samples were then shipped in 10% neutral buffered formalin to StageBio in accordance with Georgia Tech’s environmental health and safety policies. At StageBio, all samples were processed in a series of alcohol and xylene and embedded in paraffin wax for sectioning. Two 5-μm sections were produced from the center of each segment. One section was stained with Hematoxylin and Eosin (H&E) and the other was stained with Gomori’s Elastin Trichrome (GET).

The tissues were evaluated by Dr. Serge Rousselle, a pathologist at StageBio. Raw data tables were created that contained scores for multiple damage morphologies for each tissue segment. A description of the damage morphologies that were considered can be seen in the full pathologist report in the Supplementary Material. After the raw data tables were created, the pathology lab was sent information to match each segment with a wavelength and dosage. The scores in the raw data table were compiled by irradiation condition, and a report was generated that identified damage trends based on the wavelength and dosage. The level of baseline damage in negative controls (not irradiated) was also considered by the pathologist when evaluating the tissue.

### 2.4 Statistics

The initial evaluation by the pathologist was done on a categorical scale, so the data were tested for significance using Brown-Forsythe with Games-Howell as the *post hoc*. Scores for each tissue segment were treated as an independent data point. Each wavelength (1064 and 1720 nm) was tested separately. To compensate for the increased likelihood of type I errors, the significance threshold was reduced by a factor of 2 (p<0.025). All statistical tests were performed in IBM SPSS Statistics.

## 3 Results

Multiple morphological changes were identified and evaluated by the pathologist. The different morphologies were scored using a numeral categorization scale that ranged from 0 to 4 (Table 2) for each tissue segment. The score for each change and every tissue segment is reported in the tabulated microscopic data section at the end of the pathology report in the Supplementary Material. All the observations that received a nonzero damage score in at least some of the samples are shown in Table 3. The types of damage indicated by these observations were endothelial cell loss, hypereosinophilic smooth muscle cells, compressive/pressure necrosis/cell effacement, contraction bands, necrotic/apoptotic cells, and collagen denaturation. A fuller description of these observations can be found in page 3 of the pathology report in the Supplementary Material.

**Table 2:** Scoring method used by the pathologist.

Score	Description
0	Finding not present
1	Present, but minimal feature
2	Notable feature; mild
3	Prominent feature; moderate
4	Overwhelming feature; severe

**Table 3:** Damage morphologies identified by the pathologist with nonzero damage scores in at least some of the histological images.

Damage grouping	Damage endpoint
Injury	Mural acute thermal injury
	Media necrosis
	Hyalinized collagen without other thermal injury
Inflammation	Inflammation mean
	Inflammation median
	Neutrophils
	Inflammation, adventitia
Healing	Endothelialization
	Endothelium erosion (terminal or artifact)
	Hemosiderin/hemorrhage, vessel wall
	Adventitia edema

**Fig. 2:**
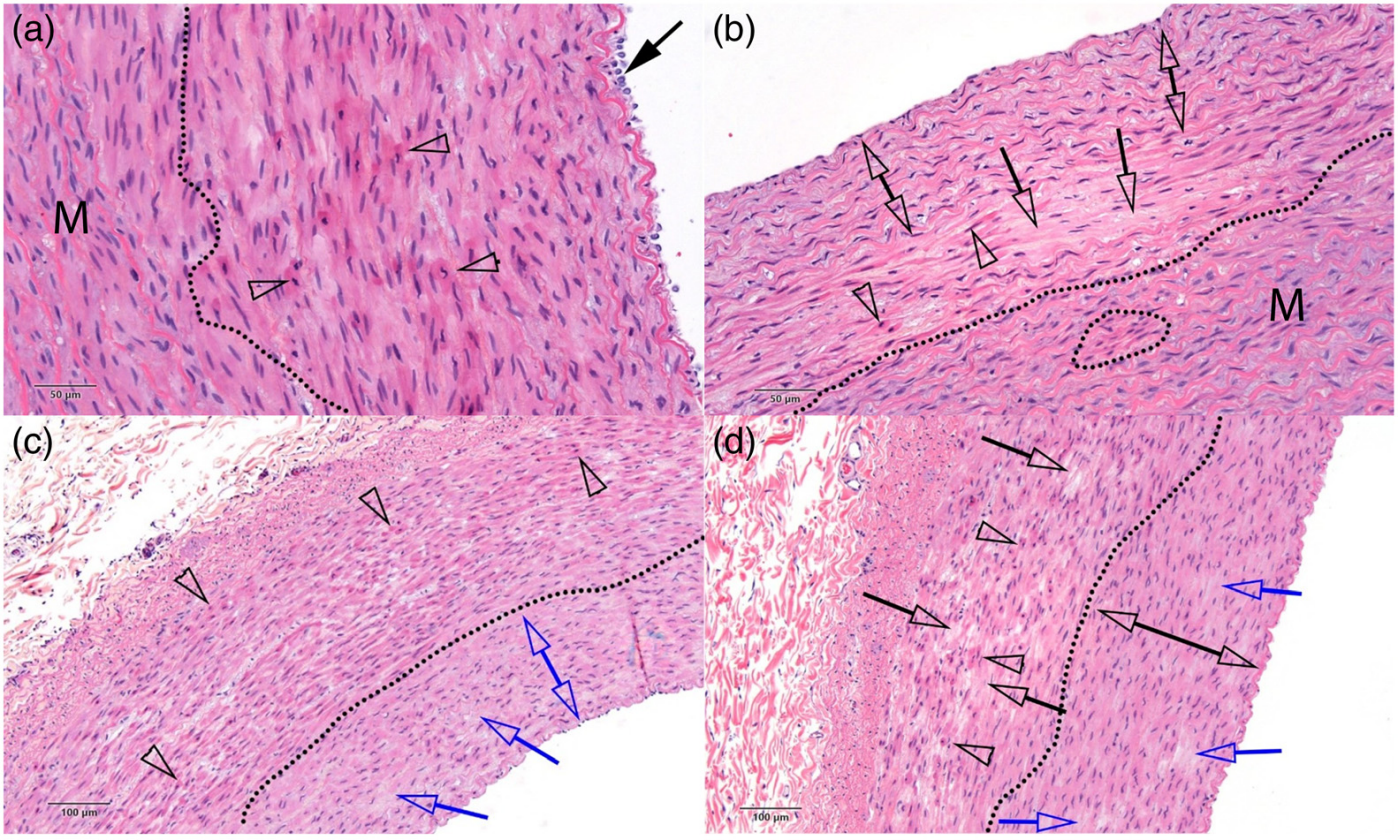
Examples of carotid artery vessels subjected to various dosages of light radiation after staining with H&E. The dosages by panel are (a) negative control, no irradiation; (b) 1064 nm, $8.3\;J/{\mathrm{cm}}^{2}$; (c) 1064 nm, $100\;J/{\mathrm{cm}}^{2}$; and (d) 1064 nm, $700J/{\mathrm{cm}}^{2}$. The dotted lines separate intact regions (M) of vessel tissue from damaged regions. Clear arrowheads indicate hypereosinophilic and contracted SMCs. The blue and black single arrows show regions of SMC effacement/loss. The double blue area in (c) shows pyknosis of SMCs from compressive necrosis, whereas the double black arrow indicates intact region of vessel in (d). The solid black arrow in (a) shows some intact media. The trend shows a nonzero baseline of damage in the control condition, with the incidence of damage increasing at higher light dosages. Additional notated images of samples subjected to these dosages are shown in the pathology report in the Supplementary Material.

**Fig. 3:**
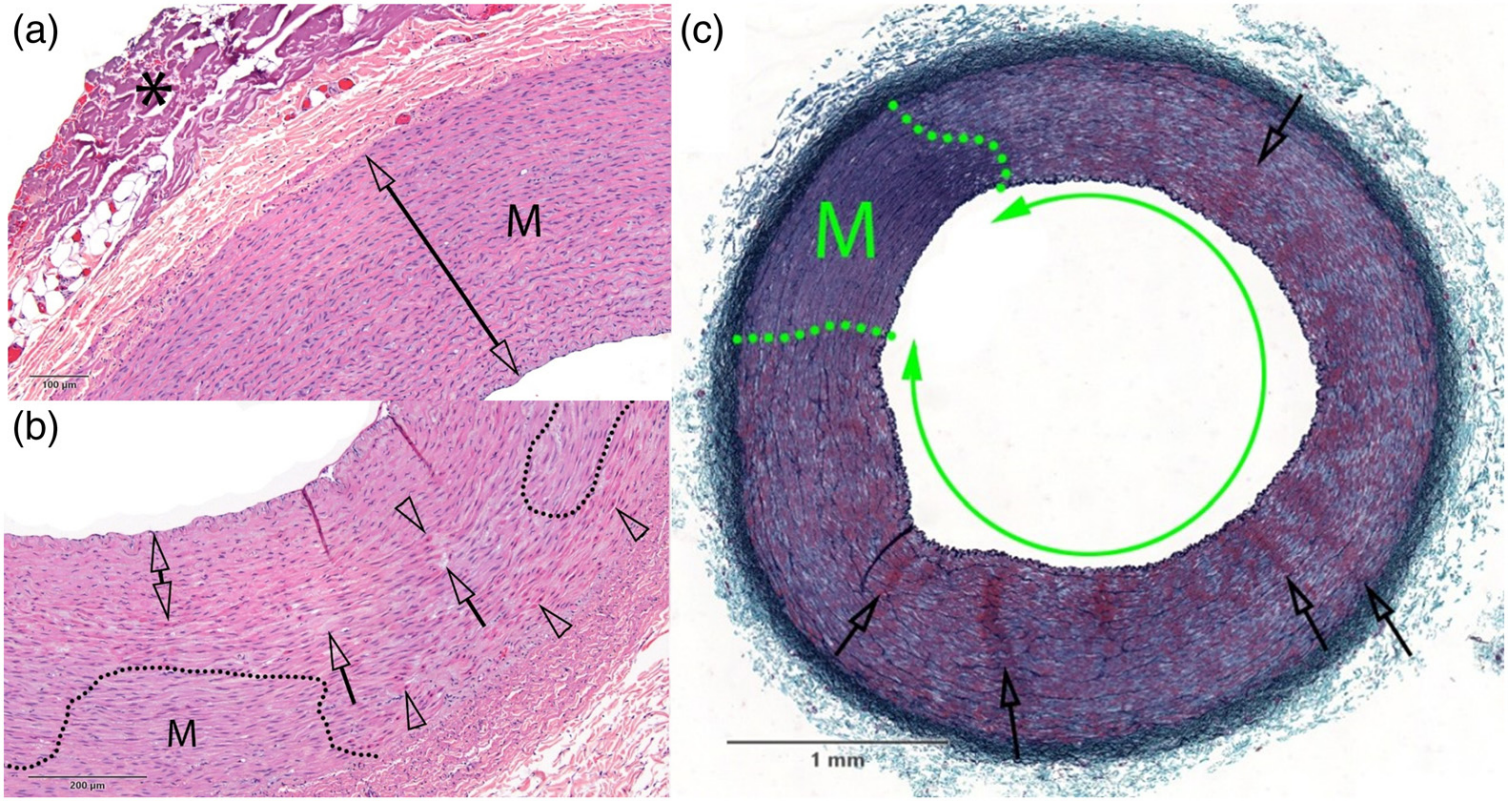
Examples of carotid artery vessels subjected to various dosages of light radiation after staining with (a), (b) H&E and (c) GET. The dosages by panel are (a) 1720 nm, $8.3\;{J/\mathrm{cm}}^{2}$; (b) 1720 nm, $50\;J/{\mathrm{cm}}^{2}$; and (c) 1720 nm, $200\;J/{\mathrm{cm}}^{2}$. The dotted lines separate intact regions (M) of vessel tissue from damaged regions. The asterisk in (a) marks collagen denaturation, likely from electrocauterization used to remove tissue during the surgery. Otherwise, no damage was detected in (a). In (b), clear arrowheads indicate hypereosinophilic and contracted SMCs, whereas clear arrows show regions with cell effacement. The double arrow indicates a region with pyknotic nuclei and hypereosinophilic SMCs, consistent with compressive injury. Damage is present throughout the vessel in (c), in the form of widespread media necrosis (double green arrow). In addition, clear arrows show radial clusters of hyperchromatic and shrunken SMCs evoking contraction bands. Scale bar shows physical size of carotid artery. Additional notated images of samples subjected to these dosages are shown in the pathology report in the Supplementary Material.

Twenty-six images of the 48 tissue segments are shown in the report with annotations and a description of any damage found by the pathologist in the Supplementary Material. One of these images is shown for each light dosage condition as an example (Figs. 2 and 3), with the annotations from the pathologist changed for formatting consistency only. Figure 2 has a sample from the negative control and each of the 3 conditions at 1064 nm. Figure 3 shows one image from each of the 3 conditions at 1720 nm. The reader can see additional images in the Supplementary Material, which contains the full pathologist report. Compressive necrosis, collagen denaturization in the adventitia, and endothelial cell loss were present at all conditions, including the negative control. With increasing severity, hypereosinophilic smooth muscle cells, cell effacement, and necrotic/apoptotic cells were present in the tissue as the radiation dosage increased. At the highest dosage at 1720 nm, contraction bands were also present.

The pathologist determined that damage was present in the tissue at clearly detectable levels for only two of the eleven microscopic observations recorded. Those observations were endothelial cell loss and media necrosis. The average pathologist scores for each of these damage morphologies are shown in Table 4. Endothelial cell loss was scored at the highest possible grade (4.0) for every tissue condition. The average scores for media necrosis ranged from 0.5 to 3.3. Although some media necrosis was present in smooth muscle cells at all conditions (an expected result of tissue handling), the severity and extent of the damage increased at higher dosages. Media necrosis was determined by the pathologist to clearly exceed the baseline damage found in the negative controls for conditions C5 and C6. Damage was also found to exceed the baseline damage in the negative controls for condition C3, although the pathologist noted that the damage was more equivocal (a trend) when compared with the negative controls.

**Table 4:** Average scores for endothelial cell loss and media necrosis. The damage for endothelialization was significant for all conditions, which is common for all catheterization procedures. The media necrosis score showed increased severity as the light dosage was increased.

Condition number	Wavelength (nm)	Fluence ($J/{\mathrm{cm}}^{2}$)	Endothelialization	Media necrosis
C0	None	0	4.0	0.7
C1	1064	8.3	4.0	1.1
C2		100	4.0	0.7
C3		700	4.0	1.4
C4	1720	8.3	4.0	0.5
C5		50	4.0	2.2
C6		200	4.0	3.3

The statistical evaluation of the media necrosis scores matched the observations of the pathologist. For the 1720-nm wavelength, the Brown-Forsythe test indicated a significant difference between groups (p<0.001). The *post hoc* test between the control and treatment groups indicated a significant difference from the control for conditions C5 (p=0.0017) and C6 (p=0.00027) but not C4 (p=0.989). At 1064 nm, the Brown-Forsythe test did not indicate a significant difference between groups (p=0.240). The Games-Howell *post hoc* showed a p value of 0.198 between C0 and C3, again indicating no significant difference.

## 4 Discussion

The pathologist identified endothelial cell loss and media necrosis as the only two findings with sufficient variance from controls to be clearly observable in the exposed samples. Endothelial loss was scored at the highest severity of 4.0 for each sample. This was expected. Catheterization regularly causes deendothelialization due to the shear stress of moving the catheter along the vessel wall (pathology report in the Supplementary Material). This is a transient effect of all catheterization procedures and is known to heal and recover rapidly. Thus, in and of itself it does not present a safety concern that would preclude the use of IVPA imaging in the clinic. The second observation that was deemed significant was media necrosis. This was found in smooth muscle cells in the vessel media. Significantly, the severity increased as the radiation dosage increased (Table 4). The pathologist observed a clear treatment effect at conditions C5 and C6 (1720 nm; 50 and $200\;J/{\mathrm{cm}}^{2}$). An equivocal treatment effect (a trend) was also observed by the pathologist for condition C3 (1064 nm; $700\;J/{\mathrm{cm}}^{2}$). The pathologist determined that all detected media injury would likely heal and not be life threatening, although it would take studies with longer time points (1 to 2 months) to ascertain recovery and healing conclusively.

The results of the statistical tests on the media necrosis scores were mostly consistent with these observations. Significant differences in media necrosis compared to the control condition were clear for C5 and C6. However, a significant difference was not identified between the control and any tissue irradiated at 1064 nm. At C3, the pathologist reported some damage, although it was equivocal (indicative of a trend) given the baseline level of damage in the control tissue. The p value between C0 and C3 was 0.198. Given the low sample size (n for C3=5), a nonsignificant result could be due to low statistical power. Since this is a safety study meant to identify damage, false positives are preferable to false negatives. Thus it is conservative to assume that increased media necrosis was identified for condition C3. However, future studies with higher statistical power would be needed to confirm this.

There were several limitations and assumptions that affect the interpretation of this study. First, the control groups had multiple sources of damage. These sources include catheterization (endothelial loss and compressive trauma due to catheter bulk and rigidity), electrocauterization to remove tissue during surgery (causing adventitial collagen denaturation/hyalinization), the physical forces applied during removal and handling of tissues, and cutting the tissues into segments after removal. In addition, the time length from tissue irradiation to tissue removal and fixation was only a few hours, meaning there was relatively little time for damage to manifest. All these factors made it more difficult for the pathologist to identify damage as having been caused by irradiation rather than resulting from the procedure. Nonetheless, the pathologist was still able to draw conclusions on the basis of blinded evaluation. Second, the amount of time it took to deliver the dosage varied for each condition. Since heat will dissipate over time, a longer irradiation time could understate the damage that may occur under shorter delivery conditions with the same amount of energy. The amount of time one location in the tissue was irradiated can be calculated using the spot size of incident light on the vessel wall in the axial direction and the catheter pullback rate. The spot size can be calculated using geometry. The length of the optical fiber in the axial direction of the artery is ∼0.6  mm since it had a diameter of 0.3 mm and was polished at a 37-deg angle. Also, the half angle of light exiting the optical fiber would be ∼9.5  deg, based on the NA of the optical fiber. Given a maximum artery radius of 2.5 mm measured during the experiment, the spot size is ∼1.44  mm in the axial direction along the artery. Based on the pullback rates for each dosage, this yields irradiation times of 1, 11, 77, 3, 16, and 66 s for each condition, respectively. Time periods of 20 s or less (C1, C2, C4, and C5) would be unlikely to affect damage since it is not a significant time length for heat transfer. However, it is possible that the level of damage at conditions C3 and C6 were underestimated due to the length of time it took to apply these dosages in tissue. Future work could address this, although it would require higher power lasers than any that have been currently reported for real-time IVPA imaging.^1^^,^^4^^,^^11^ Shrinkage of the vessel due to vasospasm during this time period could have reduced the diameter of the vessel tissue for some conditions. This at most could have changed the reported fluence by twofold since the vessels had a starting diameter near 4 mm but would not have been able to reduce beyond the diameter of the polyethylene sheath, which was 2 mm. Third, although the vessel was isolated and flushed, it is likely that at least some residual blood was present in the blood vessel. Although this would have lowered the actual light dosage that reached the tissue compared to the reported dosages, it is similar to clinical conditions in which the blood cannot be removed completely. Our experiment would still overestimate damage because vessel isolation would not be conducted during clinical IVPA imaging. Instead, IVPA imaging would be completed with unblocked vessels, likely using a bolus injection of saline or other agent to temporarily displace blood as is done during IV-OCT imaging. This flowing fluid would also exert a convective cooling effect on the blood vessel, potentially decreasing damage from heat accumulation.

The damage thresholds for media necrosis found in this study compare favorably with our previously published work on cells.^25^ In those studies, some cell types first showed statistically significant loss of viability at $690\;J/{\mathrm{cm}}^{2}$ when irradiated with light at 1064 nm. This is consistent with the results of this *in vivo* safety study, in which a trend of damage was detected at 1064 nm for dosages of $700\;J/{\mathrm{cm}}^{2}$. The IVPA lipid imaging wavelength used in the cell study was 1197 nm. At this wavelength, cell death was first apparent for some cell types at $140\;J/{\mathrm{cm}}^{2}$. In this study, damage was clear at $50\;J/{\mathrm{cm}}^{2}$ using 1720 nm light. This is 2.8× smaller than the *in vitro* threshold of $140\;J/{\mathrm{cm}}^{2}$. However, the absorption coefficient of water and vessel tissue is anywhere from 2 to 5× larger at 1720 nm than at 1197 nm,^28^^–^^30^ so a lower damage threshold at 1720 nm would be expected since more heat would be absorbed given the same light dosage. These results are in good agreement considering the large differences between the *in vitro* and *in vivo* heat transfer environments.

It is also worth comparing these results to the ANSI Z136.1 guideline for the safe use of lasers. Although this standard applies to skin and ocular tissues, we can compare our results to the requirements for skin to determine if the results seem reasonable. The maximum permissible exposure (MPE) as defined by the standard varies for each wavelength depending on the time length of irradiation. For time lengths below 10 s, it is defined in terms of the fluence applied to the tissue. For time lengths above 10 s, it is defined in terms of the irradiance applied over that time. The actual fluence applied in our experiments is already given for each condition in Table 1. The irradiance can be calculated by dividing the fluence at each dosage by the length of time it took to apply that dosage to a given region of tissue.

A comparison of the experimentally applied fluence, experimentally applied irradiance, and the MPE for each condition are shown in Table 5. Table 5 also shows if the dosage exceeded the MPE defined by the ANSI standard for each condition. The MPE has a safety factor of 10, so it must be multiplied by 10 before being compared to the experimental dosage. The final column of Table 5 indicates if the pathologist observed damage for each condition. The MPE predictions and pathologist observations agree, except that our experiments identified damage at condition C3, which was not predicted based on the MPE. This underestimate of damage when using the MPE can be explained by the fact that the absorption coefficient of vessel tissue is higher than skin at both wavelengths,^28^^–^^30^ so damage at lower dosages should be expected. Also damage at condition C3 was considered by the pathologist to be equivocal. The statistical test at this condition also yielded no significance, albeit with low sample size at this condition. Nonetheless, these results substantiate that the ANSI standards for skin may not be adequate for vessel tissue.

**Table 5:** A comparison of the MPE predicted by ANSI Z136.1 for skin to our experimental results on blood vessel tissue. Note that the ANSI Z136.1 standard defines the MPE as 10× smaller than the dosage that will cause damage 50% of the time (EC50). Thus the MPE must be multiplied by 10 before comparing it to the applied dosage.

Condition	Wavelength (nm)	Experimentally applied fluence ($J/{\mathrm{cm}}^{2}$)	Experimentally applied irradiance ($W/{\mathrm{cm}}^{2}$)	10× MPE fluence ($J/{\mathrm{cm}}^{2}$)	10× MPE irradiance ($W/{\mathrm{cm}}^{2}$)	Expected damage from MPE (Y/N)	Damage found experimentally (Y/N)
C1	1064	8.3	—	55	—	N	N
C2	1064	—	7.8	—	10	N	N
C3	1064	—	7.8	—	10	N	Y
C4	1720	8.3	—	10	—	N	N
C5	1720	—	2.6	—	1	Y	Y
C6	1720	—	2.6	—	1	Y	Y

Our experimental results indicate a damage threshold between 8.3 and $50\;J/{\mathrm{cm}}^{2}$ at 1720 nm. Assuming that the true threshold for damage is the average of these conditions, the transition point would be at $30\;{J/\mathrm{cm}}^{2}$. Reducing by the safety factor of 10 gives an MPE near $3\;J/{\mathrm{cm}}^{2}$ at 1720 nm. An IVPA imaging protocol that used 128 A-lines per frame, 0.1 mJ per pulse, and a 2-mm/s pullback rate in a 2-mm diameter (occluded) artery would be near this MPE at $3.13\;J/{\mathrm{cm}}^{2}$. Recent real-time *in vivo* studies have successfully imaged plaque lipid using imaging protocols that used equivalent or lower exposures.^1^^,^^4^^,^^11^ This indicates that it will be possible to use IVPA imaging for lipid identification safely, although further reduction in dosages may be needed to satisfy the 10× safety factor incorporated into the MPE. Future developments may show that higher dosages can be conducted safely if the type of damage identified in this study is found to heal over time. In addition, further improvements to IVPA imaging systems, such as improvements to catheter sensitivity or the use of lower frequency (near 8 MHz instead of 30 to 40 MHz) ultrasound transducers that are more sensitive to PA signals from lipid^31^^,^^32^ may make it possible to image plaque using lower light dosages.

To the authors’ knowledge, this is the first experimental study designed to investigate the safety of IVPA imaging *in vivo*. We found that damage to vessel tissue from heat accumulation is unlikely to restrict the clinical use of IVPA imaging out of safety concerns. However, the study could be improved in several ways. First, allowing for lesion differentiation and early healing by repeating the study with termination of the swine at 3 to 7 days after irradiation, which would make it easier to differentiate between damage caused by catheterization and light radiation. Second, conducting a study in which damage is evaluated 1 to 2 months after irradiation would make it possible to assess healing and recovery from the type of damage found in our histological specimens. It should be noted, however, that such a study would involve some practical difficulties. We have found during other imaging studies that it is difficult to completely flush blood from the artery in a survival surgery, because methods of isolating the vessel will still allow some blood to flow in over time. Thus blood will partially attenuate any light reaching the vessel wall. In addition, the effect of these light dosages on blood has not been investigated. If irradiation at higher dosages has a thrombogenic effect then the pigs may suffer symptoms from blood clots before the tissue can be evaluated, particularly at the higher dosages necessary for safety studies. In addition, it would be valuable to conduct a safety study on atherosclerotic swine to determine if diseased tissue has a different threshold for damage than healthy tissue. Finally, this study did not examine the effect of irradiation on blood. Future studies would be needed to assess any dosage dependent increases in thrombogenicity.

## 5 Conclusions

To the authors’ knowledge, this is the first study designed to evaluate the safety of IVPA *in vivo*. The damage to blood vessel tissue from real-time *in vivo* delivery of light was investigated in the carotid arteries of swine. Vessel tissue was exposed to light at 1064 nm and the lipid imaging wavelength of 1720 nm at multiple dosages. Tissue damage was assessed by an experienced pathologist. The pathologist determined that the only dose-dependent type of damage in the tissue was media necrosis. There was clear damage to the tissue when subjected to $50\;{J/\mathrm{cm}}^{2}$ at 1720 nm. There was also damage at $700\;J/{\mathrm{cm}}^{2}$ when 1064 nm light was used, although damage at this condition was more equivocal when compared to the controls. An MPE under the damage threshold identified at 1720 nm could be met with an imaging protocol using 128 A-lines, 0.1 mJ per pulse, and a pullback rate of 2 mm/s in a 2-mm diameter occluded artery using unfocused light emission from the catheter as was used in this study. Prior studies have already shown that lipid plaque can be imaged using similar or lesser dosages. Moreover, the damage that was detected at the higher dosages would be expected to heal, although additional studies would be needed to prove this conclusively. Future work should evaluate any healing of damage over longer time points (1 to 2 months) and evaluate any potential increase in blood thrombogenicity from light radiation. This study suggests that it will be possible to use IVPA imaging to identify lipid in plaque in a clinical setting without damaging the vessel tissue.
