# Supplementary material for: In vivo safety study using radiation at wavelengths and dosages relevant to intravascular imaging
Source: J Biomed Opt. 2022 Jan 31;27(1):016003. doi: 10.1117/1.JBO.27.1.016003 (PMC8802906; doi:10.1117/1.JBO.27.1.016003)
Supplement: Supplementary file 1 [file JBO_027_016003_SD001.pdf]

Alizée Pathology

Office: (240) 356-0056
Fax: (866) 929-5455

| | |
|---|---|
| Alizée Pathology, LLC | Executive Summary Pathology Report |
| Alizée Project Number | HJC19-339 |
| **Study Title** | **Safety Evaluation of Swine Carotid Arteries Subjected to Intravascular Photoacoustic Imaging (IVPA) at Varying Power** |
| Histopathology Site | Alizée Pathology, LLC (Alizée)<br>[a StageBio Company]<br>20 Frederick Road<br>Thurmont, MD 21788 |
| Prepared for | Georgia Institute of Technology<br>793 Marietta Street<br>Atlanta, GA 30318 |
| Date | 3/24/2021 |

Alizée Pathology, LLC

Executive Summary Pathology Report

Safety Evaluation of Swine Carotid Arteries Subjected to Intravascular Photoacoustic Imaging (IVPA) at Varying Power

Georgia Institute of Technology

Study Number HJC19-339

## Objective

The objective of this study was to histologically evaluate the local response of Intravascular Photoacoustic Imaging (IVPA) on swine carotid arteries.

## Methods

The histology and pathology portions of Alizée Project Number HJC19-339 conducted at Alizée Pathology were neither intended nor required to be in compliance with United States Food and Drug Administration GLP regulations set forth in Title 21 Code of Federal Regulations (CFR) Part 58. As such, quality assurance inspections and oversight were not performed. However, all study conduct performed at Alizée was completed in accordance with the technical memo, study protocol, Alizée Standard Operating Procedures (SOP) and sound scientific judgment. The results from analysis provided in this report have undergone a thorough quality control review. In addition to the hard copy, an electronic copy of this report in portable document format (PDF) will be provided to Georgia Institute of Technology. The PDF is a representation of the pathology report hard copy; however, only the signed hard copy of the pathology report is considered raw data. Digital images appearing in this report are for illustrative purposes only. All pathologic diagnoses were derived from the original histological preparations.

This pathology executive summary report by Alizée presents the results of microscopic assessment of a total of forty eight (48) carotid artery segments (16 segments per artery) from swine when treated with laser irradiation at several dosages for Georgia Institute of Technology, Atlanta, GA. This study, including all surgical procedures and tissue harvests, was conducted at the Georgia Institute of Technology, under the direction of Tim Sowers. Histological processing was performed at Alizée Pathology, Thurmont, MD. Resulting slides were evaluated, in a manner blinded to treatment, via light microscopy at Alizée by Serge D. Rousselle, DVM, DACVP, Study Pathologist.

According to the protocol and client communication, a total of 3 carotid arteries from swine were assigned to this study, in which there were seven total conditions (treatments). These consisted of two wavelengths of laser light (1064 nm and 1720 nm) with 3 different dosages of light at each wavelength, as well a negative control which was not irradiated with any light. The wavelength number and light dosage (fluence) are given for each condition, as shown in **Table A**. After laser irradiation at several dosages, each artery was dissected into sixteen segments and placed in containers filled with 10% neutral buffered formalin. All collected samples were sent to Alizée for histology and microscopic evaluation.

A total of sixteen (16) segments each from 3 carotid arteries (Artery 1, Artery 2 and Artery 3, respectively) were received at Alizée, fixed in 10% neutral buffered formalin. Samples were received in good condition, defined as all seals intact with no damage to containers or shipment. Histology sample totals and treatment assignment are presented in **Table A**.

**Table A. Condition and Sample Correlation**

| Condition Number | Wavelength [nm] | Fluence [J/cm^2] | Sample Numbers |
|---|---|---|---|
| C0 (negative control) | None | 0 | 1A, 1B, 1C, 1D, 2A, 2B, 2P, 3A, 3B |
| C1 | 1064 | 8.3 | 1E*, 1F*, 1G, 1H, 2C, 2D, 2E, 2F, 2G |
| C2 | 1064 | 100 | 1I, 1J, 2H, 2I, 2J, 2K, 2L |
| C3 | 1064 | 700 | 1K, 1L, 2M, 2N, 2O |
| C4 | 1720 | 8.3 | 1M, 3C, 3D, 3E |
| C5 | 1720 | 50 | 1N, 1O, 3F, 3G, 3H, 3I, 3J, 3K, 3L, 3M |
| C6 | 1720 | 200 | 1P, 3N, 3O, 3P |

* = Treatment applied to ~¼ of vessel circumference.

The pre-trimmed carotid artery segments were processed in a series of graded alcohols and xylene and paraffin wax embedded. The resulting paraffin blocks were sectioned twice serially, with an effort to capture the center of each segment, at an approximate 5 µm thickness and mounted to slide. One slide was stained with hematoxylin and eosin (H&E) and the other with Gomori's Elastin Trichrome (GET). Microscopic evaluation of the resulting slides was conducted by Serge Rousselle, DVM, DACVP. Tabulated microscopic data are presented in **Appendix A**.

Morphology parameters were generally scored using the following semi-quantitative scale (0-4):

| Score | Description |
|---|---|
| 0 | Finding not present. |
| 1 | Present, but minimal feature. |
| 2 | Notable feature; mild. |
| 3 | Prominent feature; moderate. |
| 4 | Overwhelming feature; severe. |

Definitions and causes of damage types:

Endothelial Cell Loss: Most sensitive endpoint. Endothelial cells are highly susceptible to any mechanical shear stress or thermal effect and are typically the first cell type to be lost.

Hypereosinophilic Smooth Muscle Cells: Acute cell injury in the media. The staining alteration indicates generally a change in cytoplasmic homeostasis. This can be an artifact of tissue handling or fixation if fixation conditions are not optimal or can be a very early sign of peracute mural damage. Interpretation is based on comparison between control and treated vessels as well as pattern and distribution of the change and associated changes (context).

Compressive/Pressure Necrosis/Cell Effacement (loss): Concentric change characterized by sheets of smooth muscle cells showing hypereosinophilia and pyknotic to karyorrhectic nuclei to cytolysis (loss of cellular features) leaving the vessel wall matrix intact. This change can be radial to circumferential and is always concentric (inside out).

Contraction bands: Alternating bands of hypereosinophilic and contracted smooth muscle cells alternating with pale hypocellular or acellular areas. This change indicates excessive vasoconstrictive stress and may lead to necrosis or regeneration.

Necrotic/Apoptotic Cells: Individual cell necrosis; may present as hypereosinophilia, pyknosis, karyorrhexis, cell debris and/or apoptotic body.

Collagen denaturation: Loss of fine fibrillar texture with collagenous areas showing hyalinization (homogenous thick bundles typically hypereosinophilic and sometimes picking up hematoxylin stain (purple) to varying degrees). This change indicates heat exposure (thermal denaturation).

## Results & Discussion

Microscopic scoring of the local response of a total of forty eight (48) segments from 3 swine carotid arteries when treated with laser irradiation at several dosages showed the following:

**Table B. Histopathology Group Summary**

| HJC19-339 | | INJURY | | | INFLAMMATION | | | | HEALING | | | |
|---|---|---|---|---|---|---|---|---|---|---|---|---|
| | | Mural Acute Thermal Injury | Media Necrosis | Hyalinized Collagen without Other Thermal Injury | Inflammation Mean | Inflammation Median | Neutrophils | Inflammation, Adventitia | Endothelialization | Endothelium Erosion (terminal or artifact) | Hemosiderin/Hemorrhage, Vessel Wall | Adventitia Edema |
| C0 | n | 9 | 9 | 9 | 9 | 9 | 9 | 9 | 9 | 9 | 9 | 9 |
| | Mean | 0.0 | **0.7** | **0.3** | **0.1** | 0.0 | **0.4** | **0.4** | **4.0** | **1.8** | **0.8** | **0.1** |
| | StDev | 0.0 | **0.7** | **0.7** | **0.2** | **0.2** | **0.5** | **0.5** | 0.0 | **1.1** | **0.7** | **0.3** |
| C1 | n | 9 | 9 | 9 | 9 | 9 | 9 | 9 | 9 | 9 | 9 | 9 |
| | Mean | 0.0 | **1.1** | **0.1** | **0.2** | 0.0 | **0.7** | **0.7** | **4.0** | **1.3** | **0.9** | **0.6** |
| | StDev | 0.0 | **0.6** | **0.3** | **0.2** | **0.2** | **0.5** | **0.5** | 0.0 | **0.5** | **0.3** | **0.7** |
| C2 | n | 7 | 7 | 7 | 7 | 7 | 7 | 7 | 7 | 7 | 7 | 7 |
| | Mean | 0.0 | **0.7** | 0.0 | **0.1** | 0.0 | **0.1** | **0.1** | **4.0** | **0.4** | **0.7** | 0.0 |
| | StDev | 0.0 | **1.0** | 0.0 | **0.2** | **0.2** | **0.4** | **0.4** | 0.0 | **0.5** | **0.5** | 0.0 |
| C3 | n | 5 | 5 | 5 | 5 | 5 | 5 | 5 | 5 | 5 | 5 | 5 |
| | Mean | 0.0 | **1.4** | 0.0 | **0.2** | 0.0 | **0.6** | **0.6** | **4.0** | **1.2** | **1.0** | 0.0 |
| | StDev | 0.0 | **0.5** | 0.0 | **0.2** | **0.2** | **0.5** | **0.5** | 0.0 | **0.4** | 0.0 | 0.0 |
| C4 | n | 4 | 4 | 4 | 4 | 4 | 4 | 4 | 4 | 4 | 4 | 4 |
| | Mean | 0.0 | **0.5** | **0.5** | **0.4** | **0.5** | **0.8** | **0.8** | **4.0** | **0.8** | **0.3** | 0.0 |
| | StDev | 0.0 | **1.0** | **1.0** | **0.3** | **0.3** | **0.5** | **0.5** | 0.0 | **0.5** | **0.5** | 0.0 |
| C5 | n | 10 | 10 | 10 | 10 | 10 | 10 | 10 | 10 | 10 | 10 | 10 |
| | Mean | **0.2** | **2.2** | 0.0 | **0.2** | **0.2** | **0.6** | **0.6** | **4.0** | **1.9** | **0.6** | 0.0 |
| | StDev | **0.4** | **0.8** | 0.0 | **0.2** | **0.2** | **0.5** | **0.5** | 0.0 | **0.9** | **0.5** | 0.0 |
| C6 | n | 4 | 4 | 4 | 4 | 4 | 4 | 4 | 4 | 4 | 4 | 4 |
| | Mean | 0.0 | **3.3** | 0.0 | 0.0 | 0.0 | 0.0 | 0.0 | **4.0** | **3.5** | **1.0** | 0.0 |
| | StDev | 0.0 | **0.5** | 0.0 | 0.0 | 0.0 | 0.0 | 0.0 | 0.0 | **0.6** | **0.8** | 0.0 |

**General Perspective on Acute Vascular Changes Associated with Energy Delivery:**

Energy delivery produce non-specific microscopic changes in tissues essentially characterized by degeneration and necrosis sometimes accompanied by evidence of extracellular protein matrix denaturation. In vessels, these changes target endothelial cells and media smooth muscle cells primarily. Extracellular matrix (ECM) can be affected by mechanical or energy delivery and cause laceration or tears (mechanical stress) or coagulation (radiative energy). Spurious factors can and frequently do complicate interpretation of vascular histology. Vascular histology can be significantly influenced by experimental conditions in-vivo and at tissue harvest (tissue handling and fixation). Catheterization of a target vessel alone can and typically erodes the endothelial surface through mechanical shear stress. Catheter bulk and relative rigidity can also impart localized compressive trauma sufficient to bruise the vessel wall and cause cell death in the media. This can be aggravated by vasospasm at the time of treatment.

Tissue collection can also produce changes that mimic necrosis unless the tissue is fixed in-situ prior to dissection and excision/trimming. Tissue handling and pulling during dissection as well as trimming of target segments can also cause tissue damage that mimics compressive injury (crushing). Use of cauterizing scalpel can cause coagulation necrosis and denaturation of ECM.

Morphologically, the resulting effect on vascular cells include vacuolization, shrinkage and hypereosinophilia of the cytoplasm, pyknosis, localized cellular effacement or stretch tears in cellular sheets. Effect on ECM includes lacerations and coagulation/hyalinization (heat-induced denaturation). Interpreting the significance and cause of acute vascular changes must take into account the level of background noise as observed in concurrent untreated controls.

**Microscopic Finding Ascribable to Treatment:**

**Table C: Average Severity of Media Necrosis**

| | Media Necrosis (Average Score) |
|---|---|
| **C0** | 0.7 |
| **C1** | 1.1 |
| **C2** | 0.7 |
| **C3** | 1.4 |
| **C4** | 0.5 |
| **C5** | 2.2 |
| **C6** | 3.3 |

In this study, the only change that was clearly dose-related consisted of media necrosis. This change is characterized by areas of media hypereosinophilia with pyknosis and/or karyorrhexis. It was observed at low severity in control segments (C0) and showed a clear increase in severity, incidence and circumferential extension at highest doses (C5 and C6). In group C3, there was a slight increase in incidence and circumferential extent that was of equivocal significance biologically (possible trend) considering the level of background noise in the controls. The changes observed at lower doses (C1, C4) were consistent with background noise.

## C0 (NO ACTIVATION)

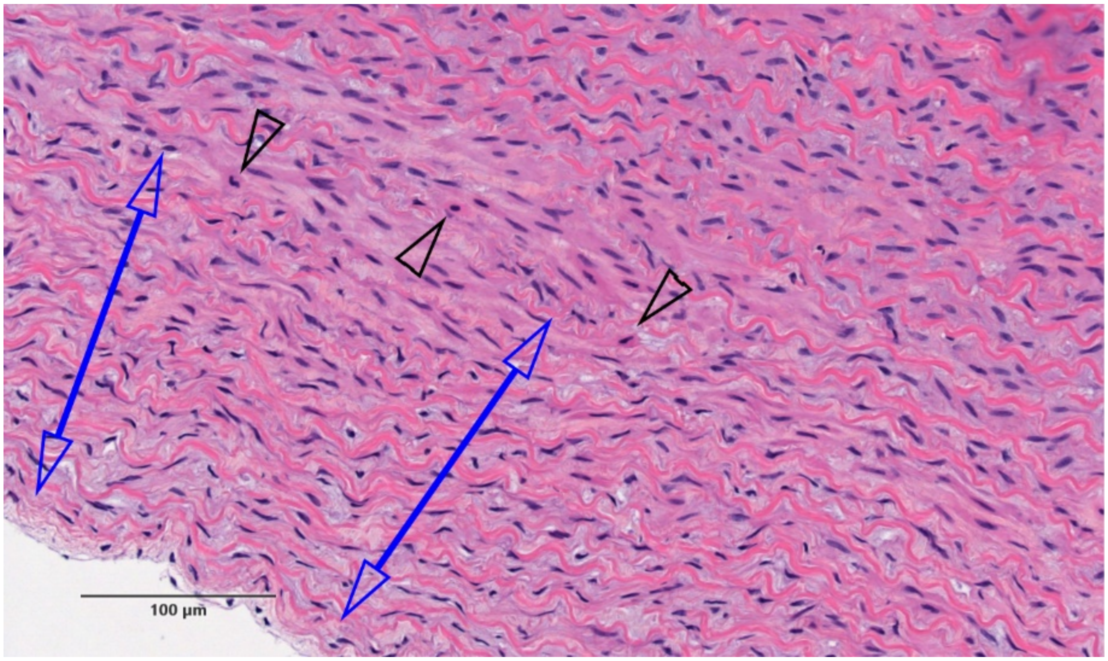


**Figure 1.** Segment 2A (H&E). C0 (No activation). Clear arrowheads = rare contracted and hyper eosinophilic smooth muscle cells in the media (media necrosis score '1'); blue double arrows = inner media showing compressed smooth muscle cells with pyknotic nuclei (compression injury).

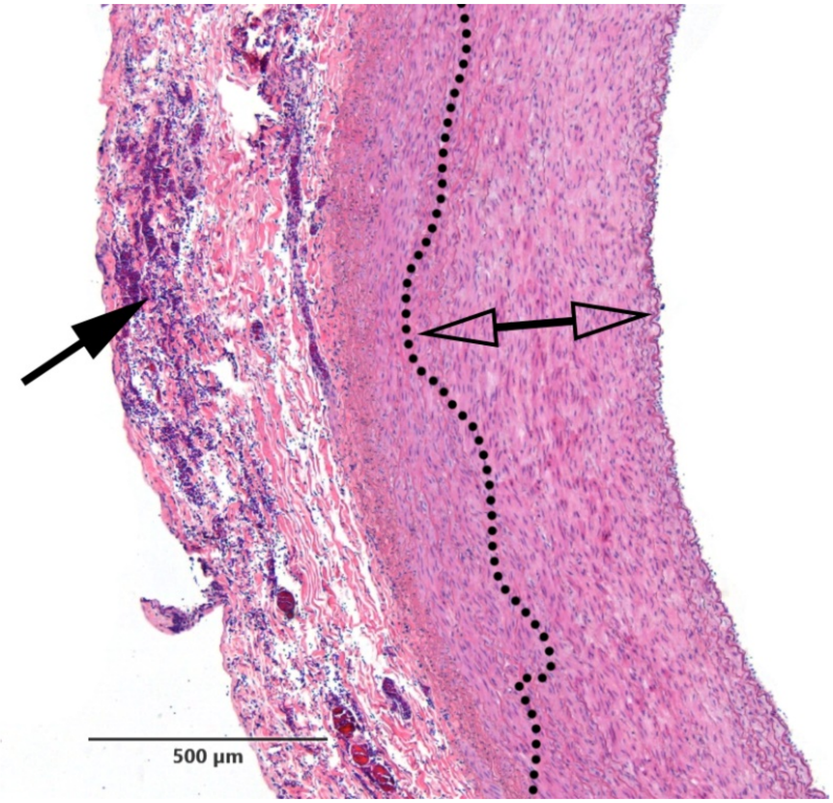


**Figure 2.** Segment 1C (H&E). C0 (No activation). Clear double arrow inside dotted line = inner media showing smooth muscle cells with pyknotic nuclei and variably hypereosinophilic and contracted cytoplasm; solid arrow = slight adventitial hermorrhage (possible collection artifact).

## C0 (NO ACTIVATION) (CONTINUED)

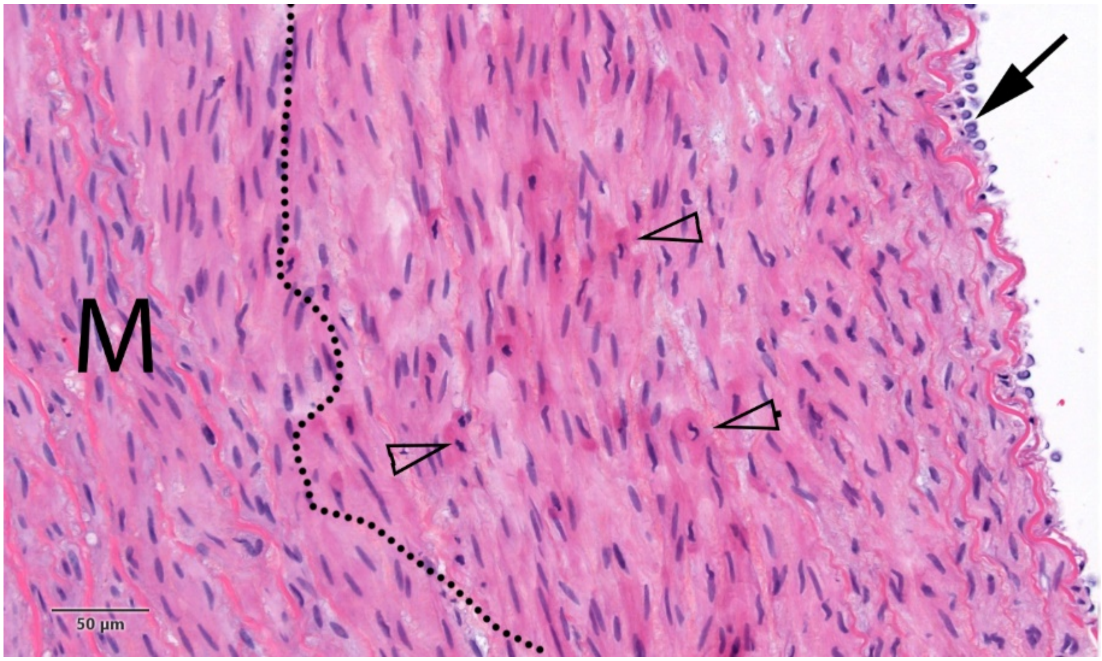


**Figure 3.** Segment 1C (H&E). C0 (No activation). M = intact outer media; black dotted line = outline of inner media area showing necrotic smooth muscle cells (clear arrowheads = hypereosinophilic and contracted smooth muscle cells); solid arrow = intact endothelium.

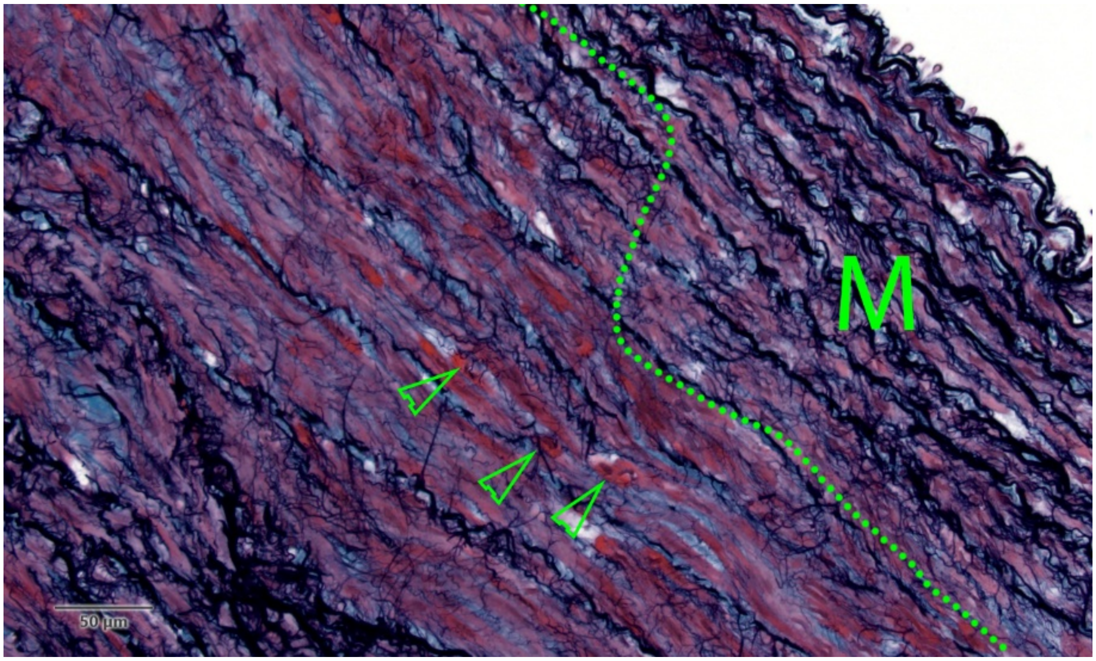


**Figure 4.** Segment 1C (GET). C0 (No activation). M = intact inner media; green dotted line = outline of deep media area showing necrotic smooth muscle cells; green arrowheads = hyperchromatic and contracted smooth muscle cells (necrosis).

## C0 (NO ACTIVATION) (CONTINUED)

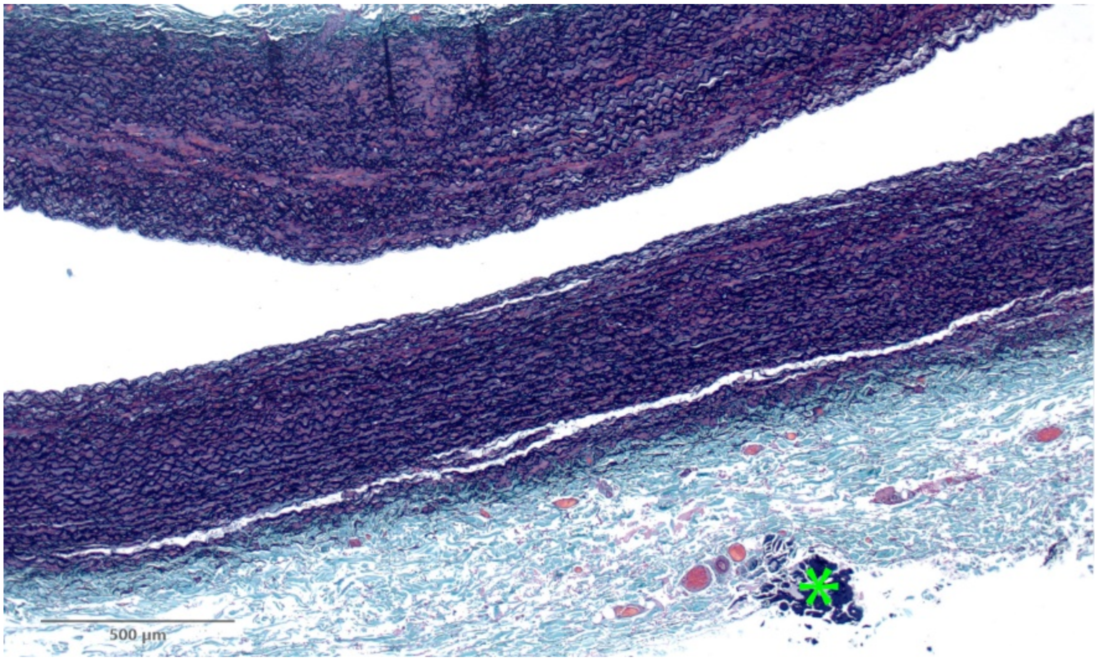


**Figure 5.** Segment 2A (GET). C0 (No activation). Green asterisk = focal collagen denaturation of the adventitial collagen (consistent with focal cauterization).

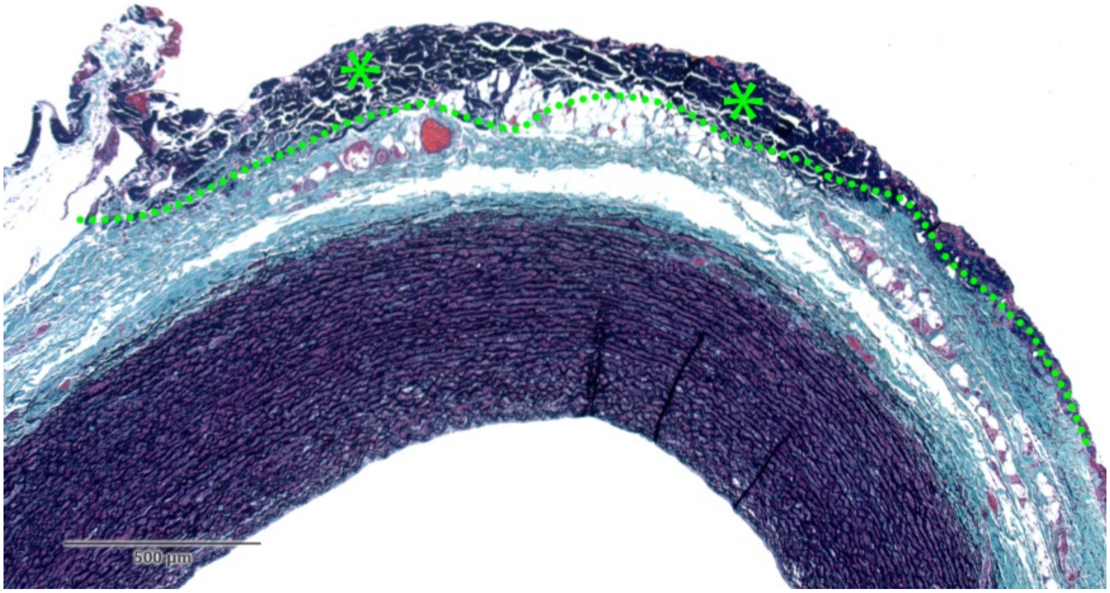


**Figure 6.** Segment 3B (GET). C0 (No activation). Green asterisks = locally extensive collagen denaturation of the adventitia (consistent with focal cauterization), delineated by green dotted line.

## C1 (1064 nm - 8.3 J/cm²)

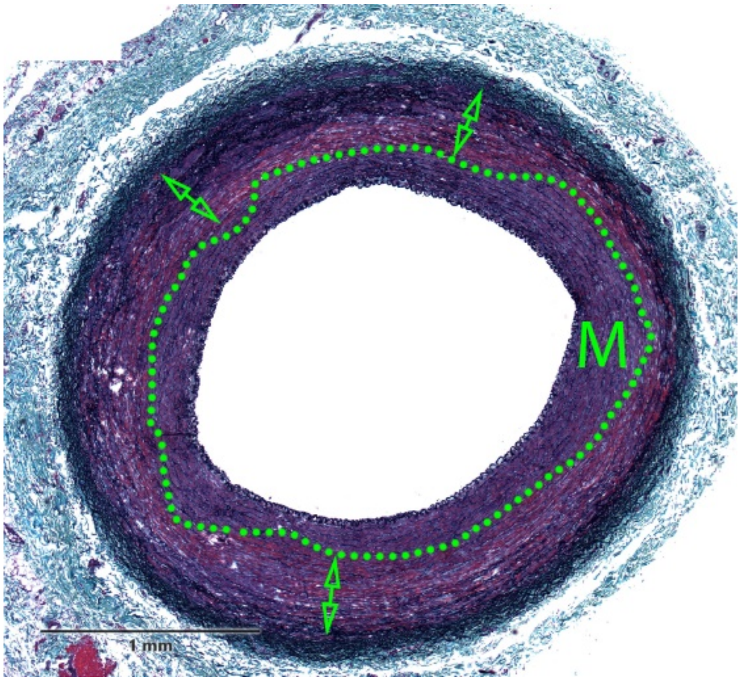


**Figure 7.** Segment 1H (GET). C1 (1064 nm - 8.3 J/cm²). Green dotted line = boundary between the intact inner media (M) and the outer affected media (green double-arrows) showing hyperchromatic smooth muscle cells.

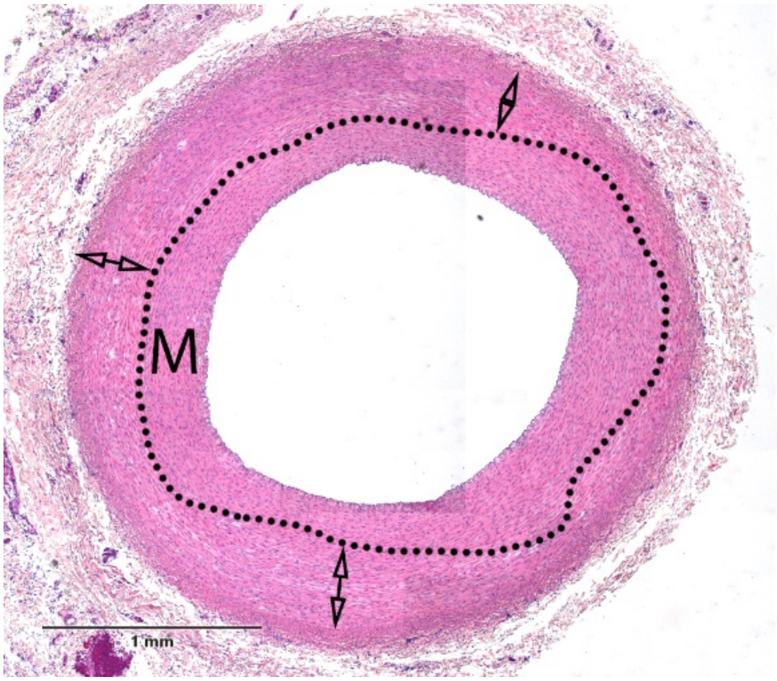


**Figure 8.** Segment 1H (H&E). C1 (1064 nm - 8.3 J/cm²). Black dotted line = boundary between the intact inner media (M) and the outer affected media (clear double-arrows) showing hypereosinophilic smooth muscle cells.

## C1 (1064 nm - 8.3 J/cm²) (CONTINUED)

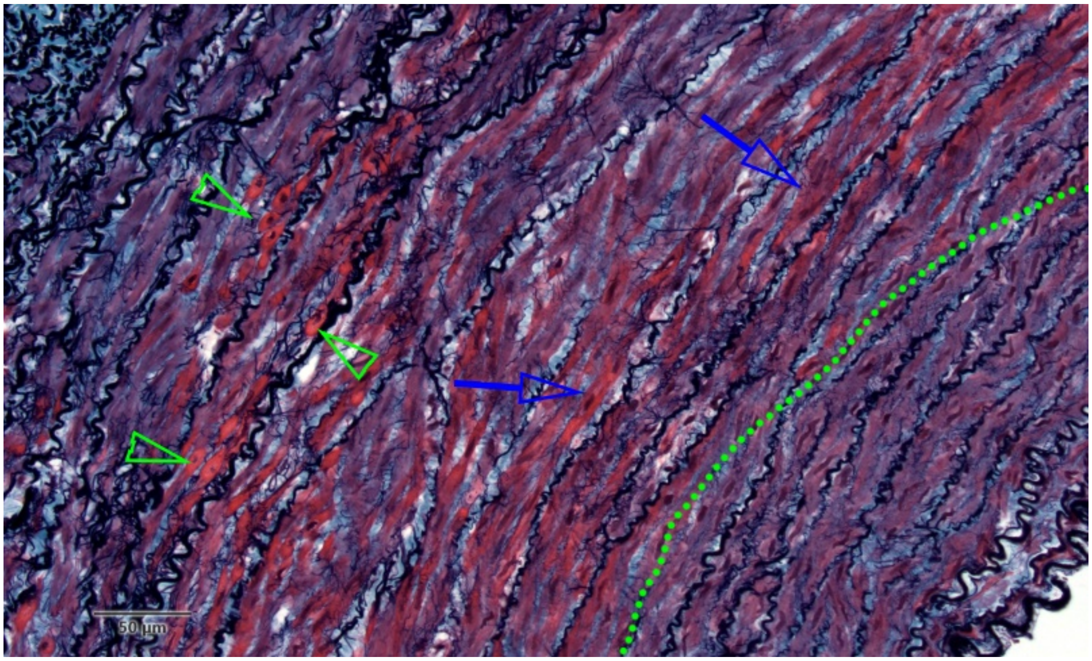


**Figure 9.** Segment 1H (GET). C1 (1064 nm - 8.3 J/cm²). Green dotted line = outline of deep media area showing necrotic smooth muscle cells; green arrowheads = hyperchromatic and contracted smooth muscle cells (necrosis); blue arrows = hyperchromatic elongated smooth muscle cells (equivocal injury).

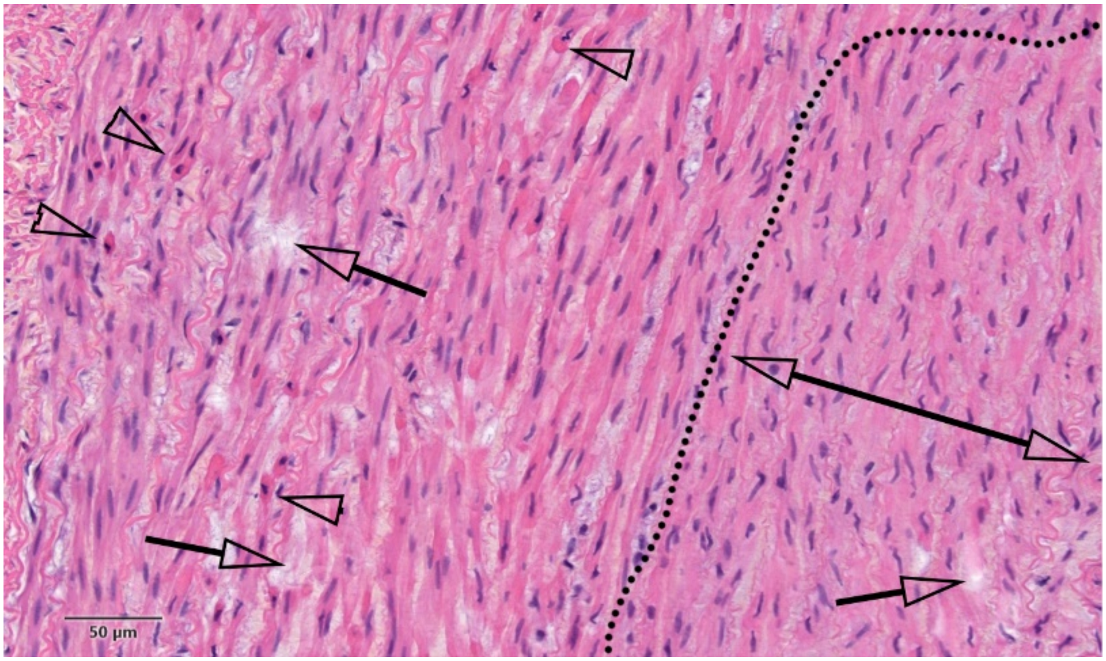


**Figure 10.** Segment 1H (H&E). C1 (1064 nm - 8.3 J/cm²). Black dotted line = outline of deep media area showing necrotic smooth muscle cells; clear arrowheads = hyperchromatic and contracted smooth muscle cells (necrosis); clear arrows = clear spaces between smooth muscle cells (edema and/or smooth muscle cell dissociation and loss); clear double arrow = inner media appearing intact, although a few clear interstitial spaces are evident (possible early damage).

## C1 (1064 nm - 8.3 J/cm$^2$) (CONTINUED)

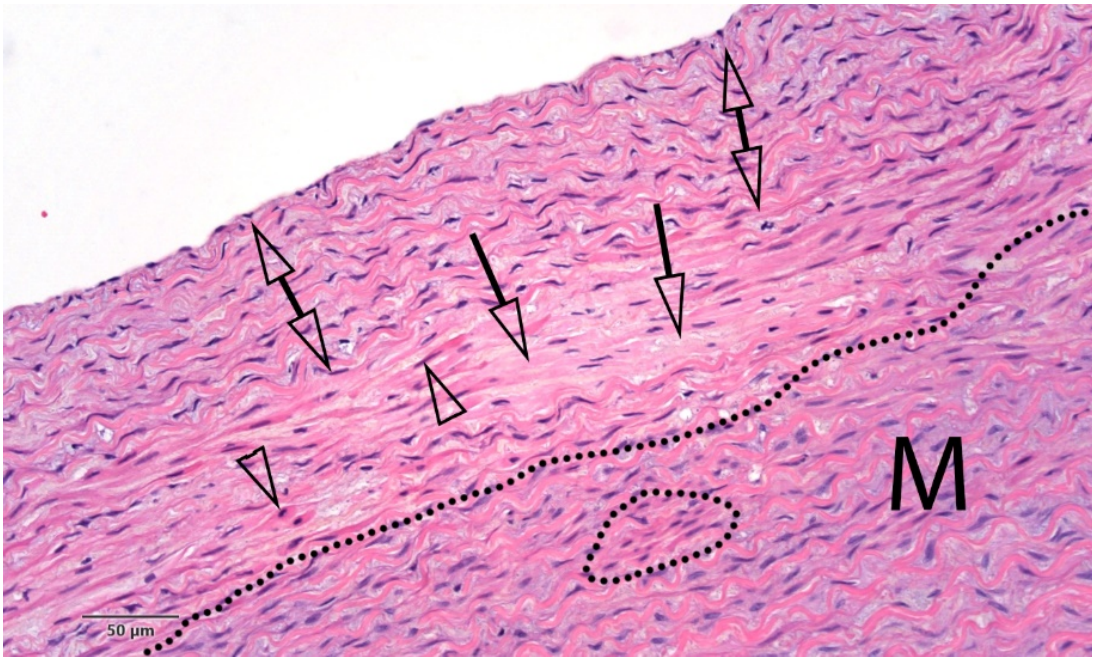


**Figure 11**. Segment 2D (H&E). C1 (1064 nm - 8.3 J/cm$^2$). Black dotted line = boundary between the intact outer media (M) and the inner affected media showing hypereosinophilic smooth muscle cells (arrowheads) and clear spaces of smooth muscle cell effacement and loss (clear arrows); clear double arrows = innermost media showing pyknosis in smooth muscle cell nuclei (generally consistent with compressive necrosis).

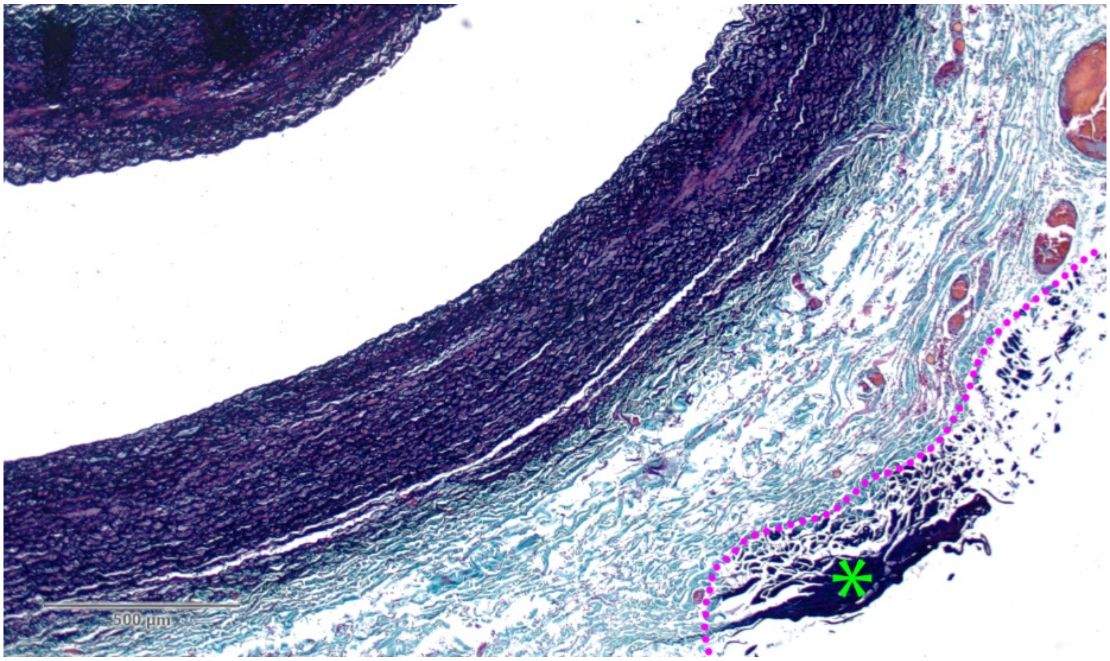


**Figure 12.** Segment 2D (GET). C1 (1064 nm - 8.3 J/cm$^2$). Green asterisk bounded by dotted line = focal collagen denaturation of the adventitial collagen (consistent with focal cauterization).

**C2 (1064 nm – 100 J/cm²)**

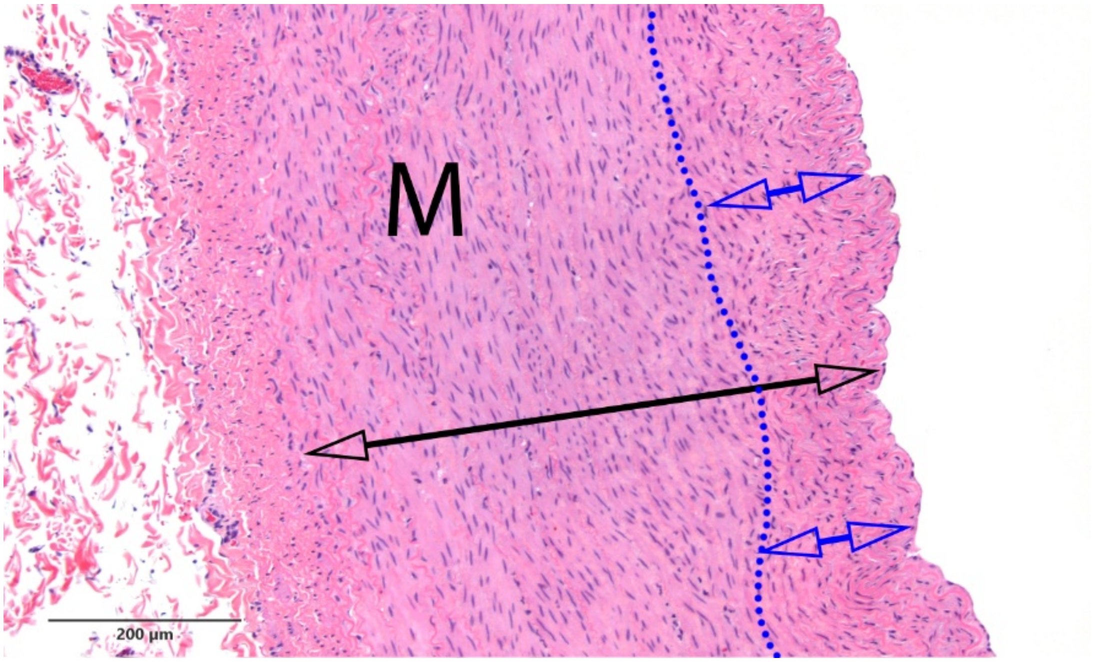


**Figure 13.** Segment 2J (H&E). C2 (1064 nm - 100 J/cm$^2$). Clear double arrow = media (M) showing no evident necrosis; blue dotted line and blue double arrows = inner media showing pyknosis on SMC nuclei, generally consistent with pressure necrosis.

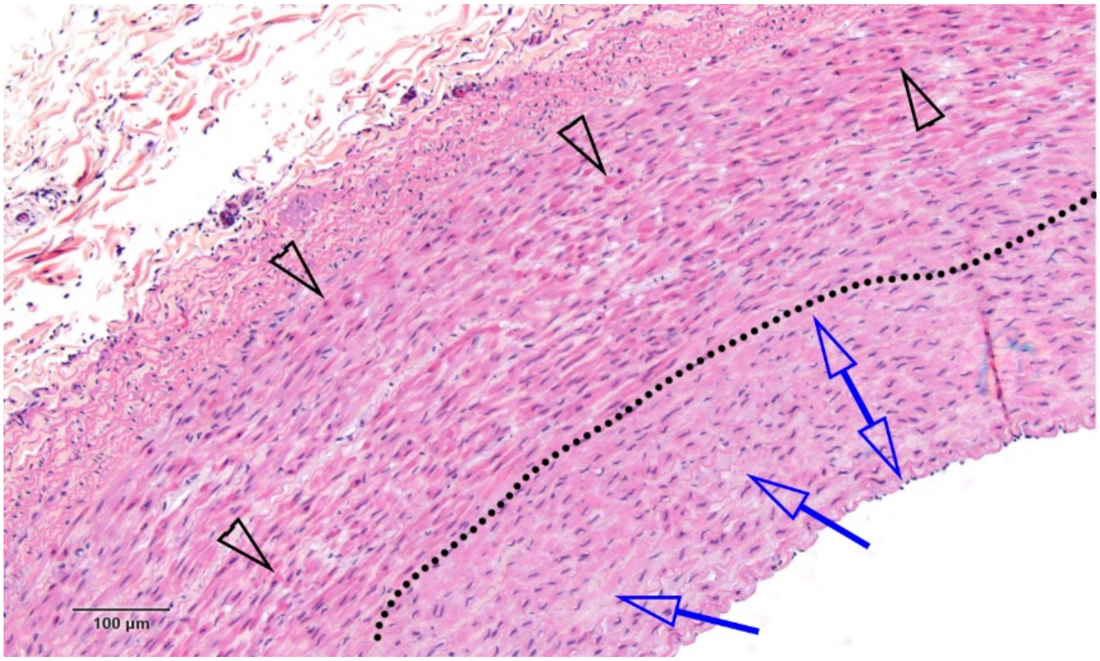


**Figure 14.** Segment 1I (H&E). C2 (1064 nm - 100 J/cm$^2$). Black dotted line = boundary between the intact outer media and the inner affected media showing hypereosinophilic smooth muscle cells (clear arrowheads) and increased interstitial clear spaces; blue double arrow = innermost media showing pyknosis in smooth muscle cell nuclei (generally consistent with compressive necrosis); blue arrows = clear spaces in the inner media (smooth muscle cell loss) denoting possible energy injury.

## C2 (1064 nm – 100 J/cm$^2$) (CONTINUED)

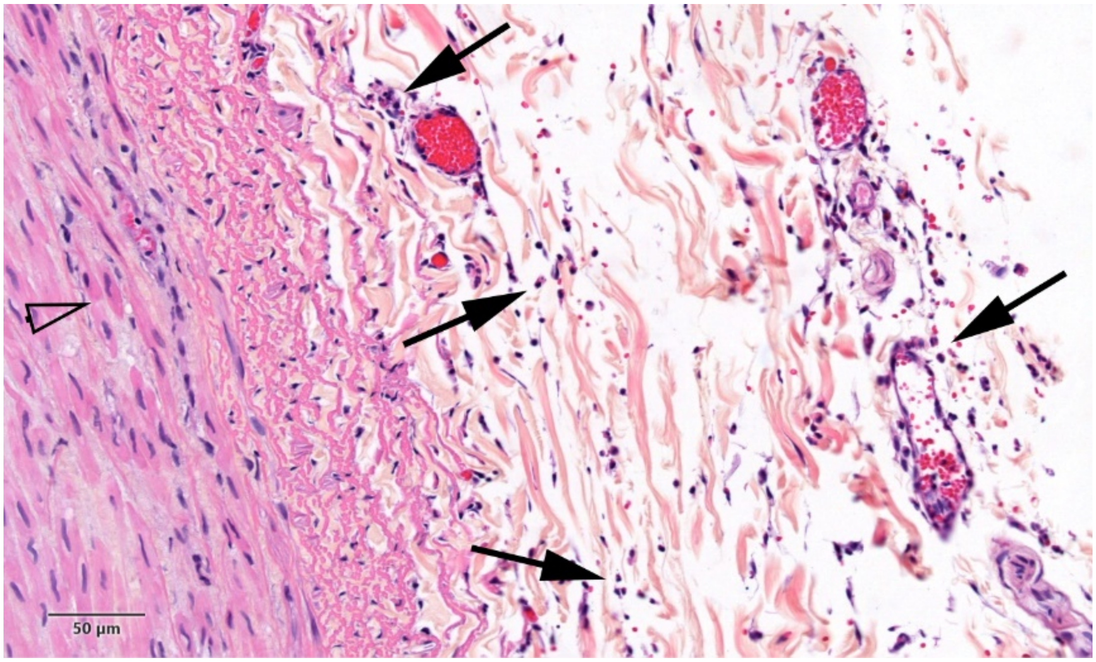


**Figure 15.** Segment 1I (H&E). C2 (1064 nm - 100 J/cm$^2$). Solid arrows = minimal inflammation in the adventitia (neutrophils); clear arrowhead = necrotic smooth muscle cells in the outer media.

## C3 (1064 nm – 700 J/cm$^2$)

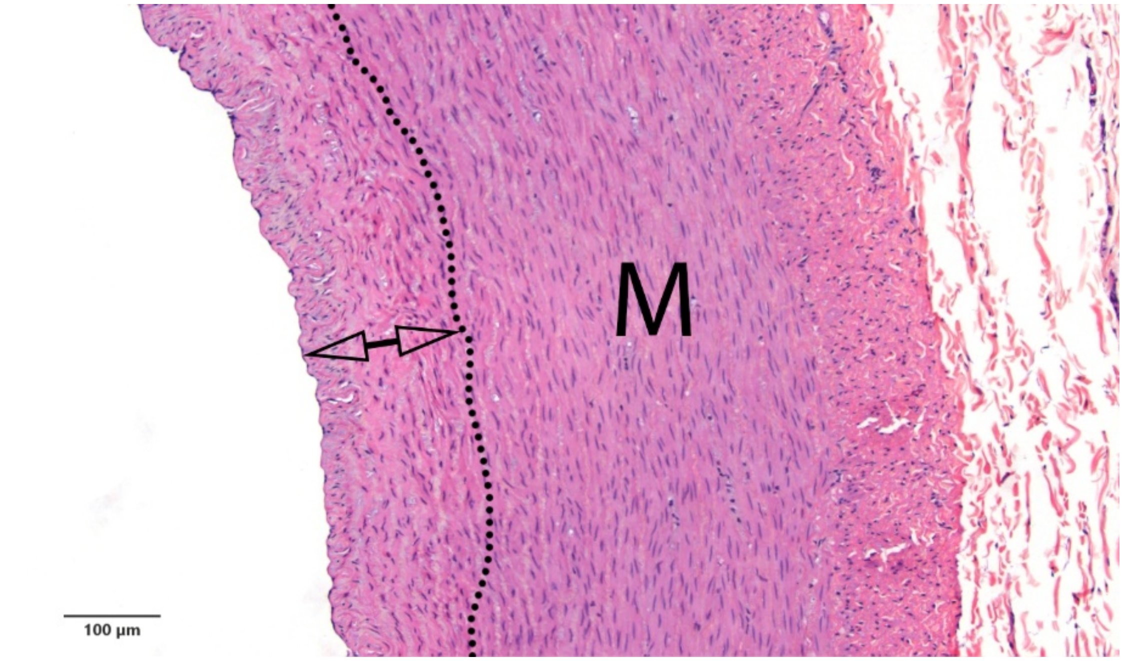


**Figure 16.** Segment 2N (H&E). C3 (1064 nm - 700 J/cm$^2$). Clear double arrow inside dotted line = media showing no evident treatment-induced necrosis. There is concentric hypereosinophilia and pyknosis typically consistent with endovascular compression; M = intact media.

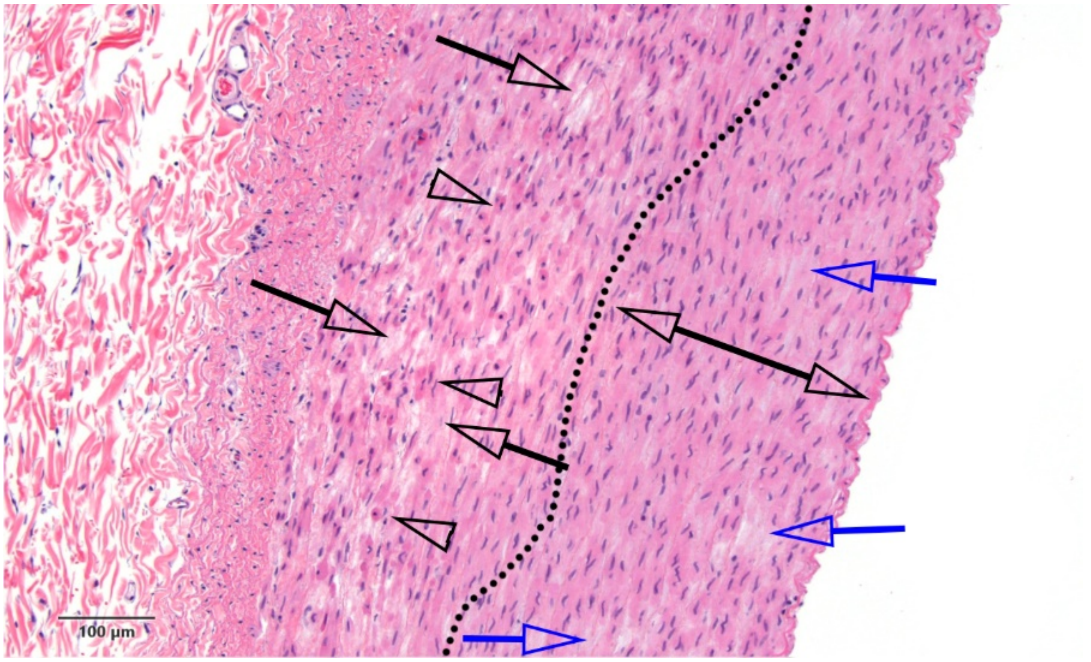


**Figure 17.** Segment 1L (H&E). C3 (1064 nm - 700 J/cm$^2$). Black dotted line = boundary between the intact inner media (clear double arrow) and the outer affected media showing hypereosinophilic smooth muscle cells (clear arrowheads) and increased interstitial clear spaces (clear arrows); blue arrows = clear spaces in the inner media (smooth muscle cell loss).

## C4 (1720 nm - 8.3 J/cm²)

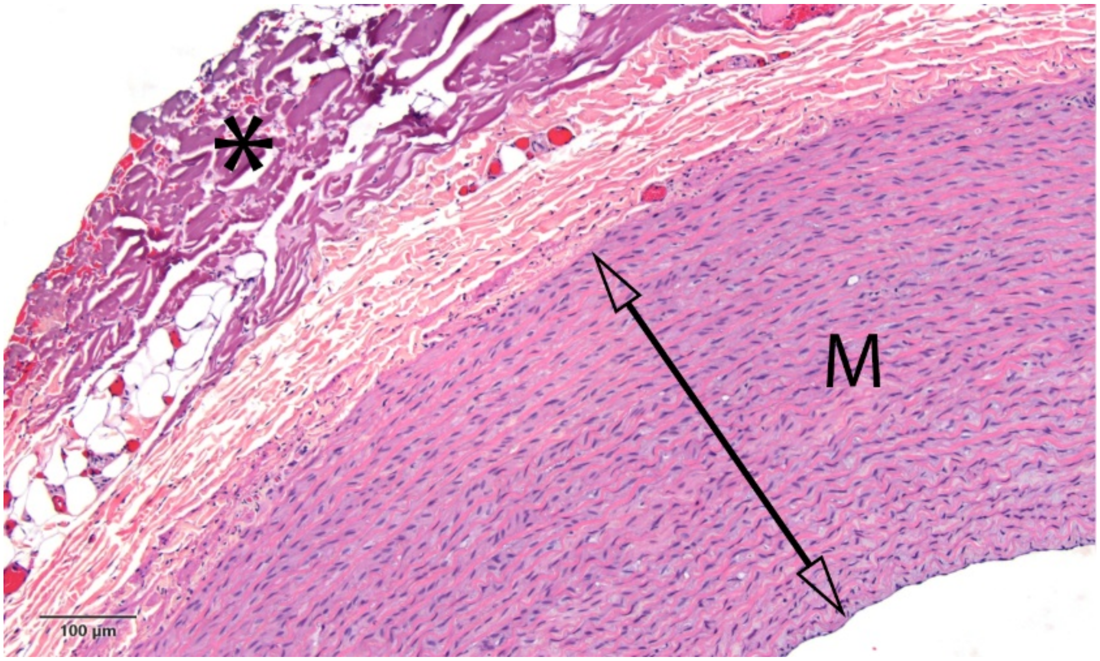


**Figure 18.** Segment 3C (H&E). C4 (1720 nm – 8.3 J/cm²). Black asterisk = collagen denaturation in the adventitia; clear double arrow = intact media (M).

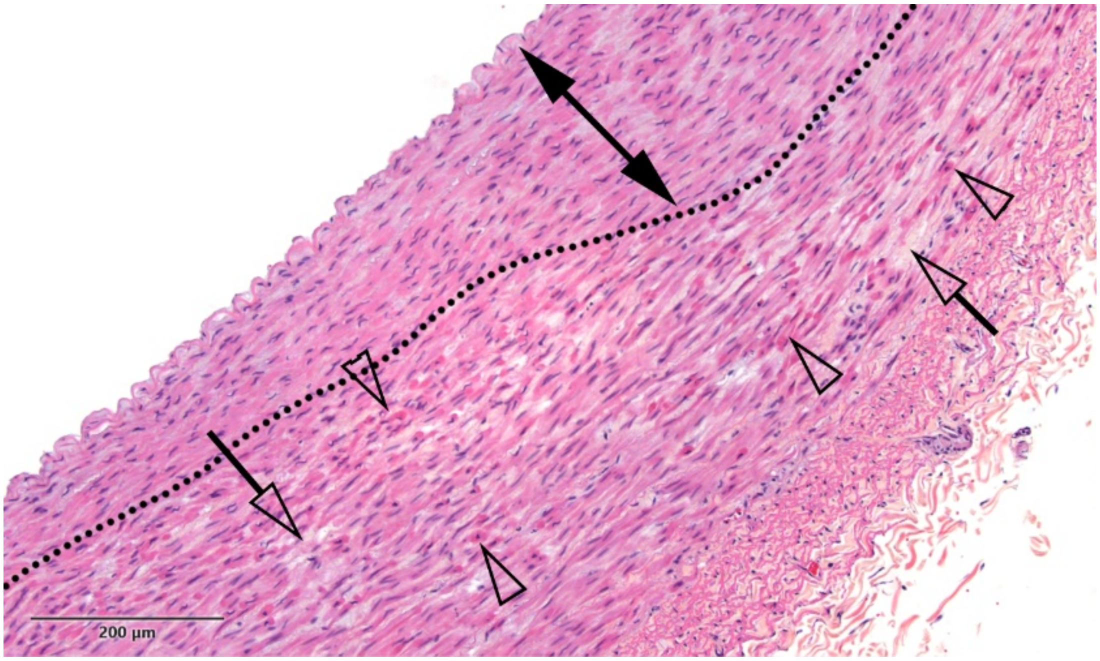


**Figure 19.** Segment 1M (H&E). C4 (1720 nm – 8.3 J/cm²). Black dotted line = boundary between the intact (albeit possibly compressed) inner media (solid double arrow) and the outer affected media showing hypereosinophilic and contracted smooth muscle cells (clear arrowheads) and increased interstitial clear spaces (clear arrows).

## C4 (1720 nm - 8.3 J/cm$^2$) (CONTINUED)

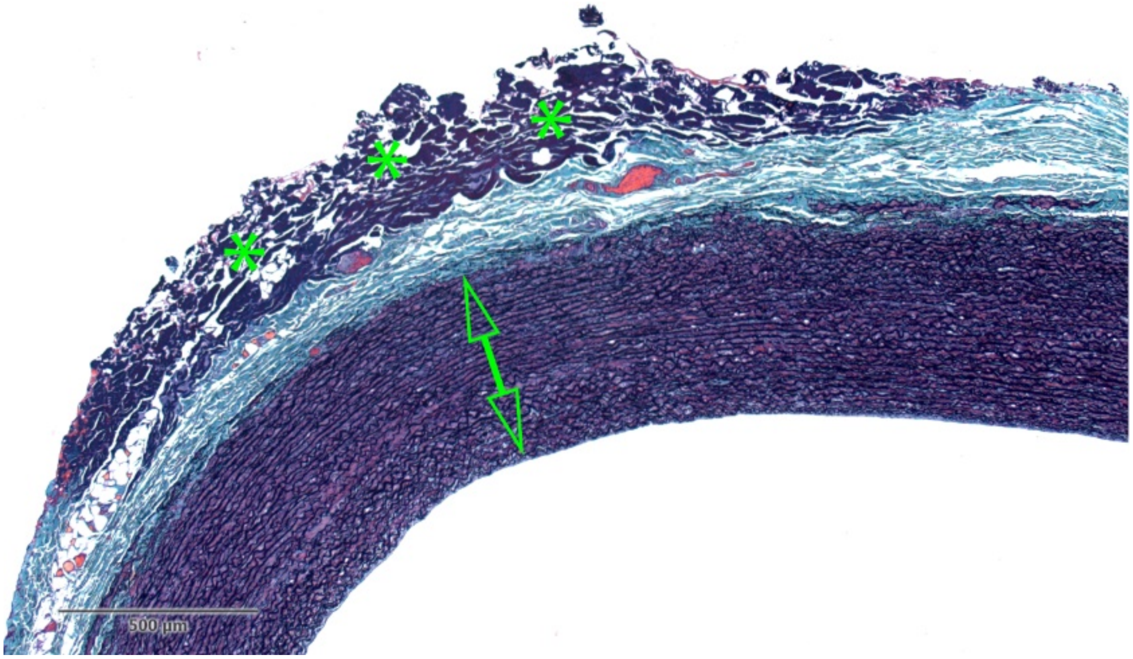


**Figure 20.** Segment 3C (GET). C4 (1720 nm – 8.3 J/cm$^2$). Green asterisks = locally extensive collagen denaturation of the adventitia (consistent with focal cauterization); green double arrow = intact media.

## C5 (1720 nm - 50 J/cm$^2$)

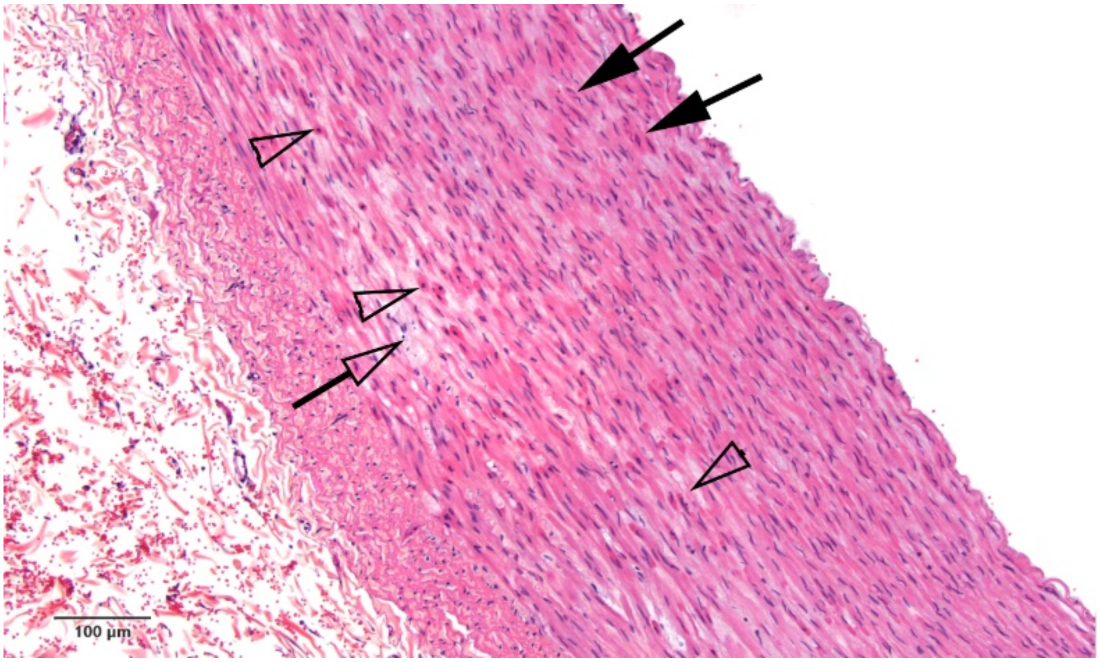


**Figure 21.** Segment 1N (H&E). C5 (1720 nm – 50 J/cm$^2$). Affected media showing hypereosinophilic and contracted smooth muscle cells (clear arrowheads) and increased interstitial clear spaces (clear arrow); solid arrows = hypereosinophilic smooth muscle cells with pyknotic nuclei in the inner media (typically consistent with compressive necrosis).

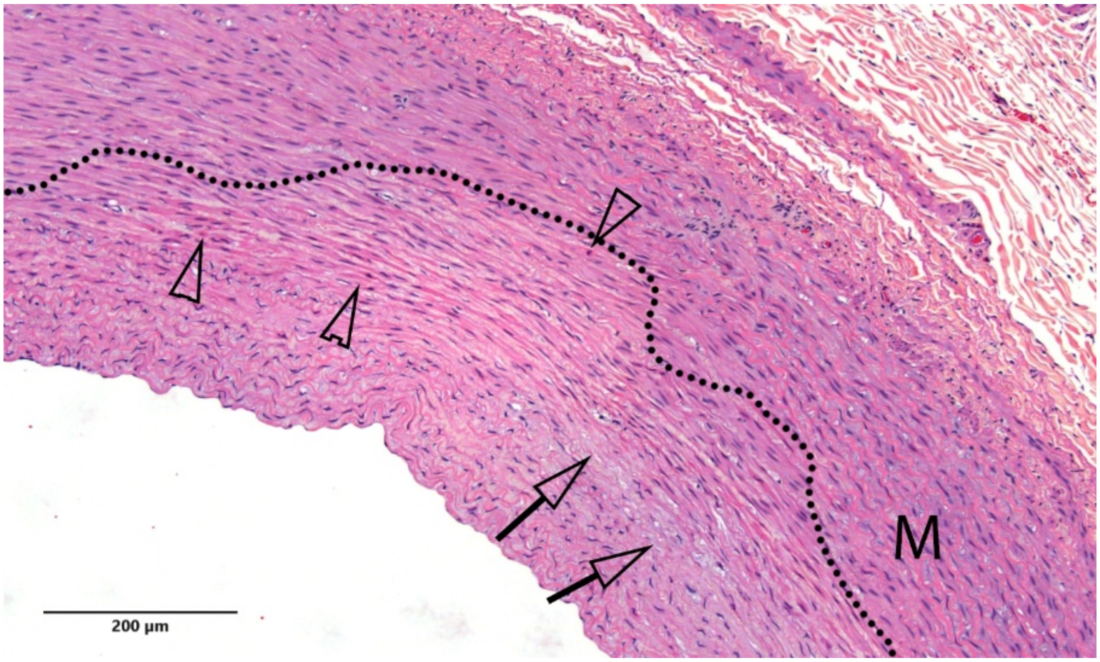


**Figure 22.** Segment 3H (H&E). C5 (1720 nm – 50 J/cm$^2$). Media inside dotted line = affected media showing hypereosinophilic and contracted smooth muscle cells (clear arrowheads) and increased interstitial clear spaces (clear arrows); M = intact media.

## C5 (1720 nm - 50 J/cm$^2$) (CONTINUED)

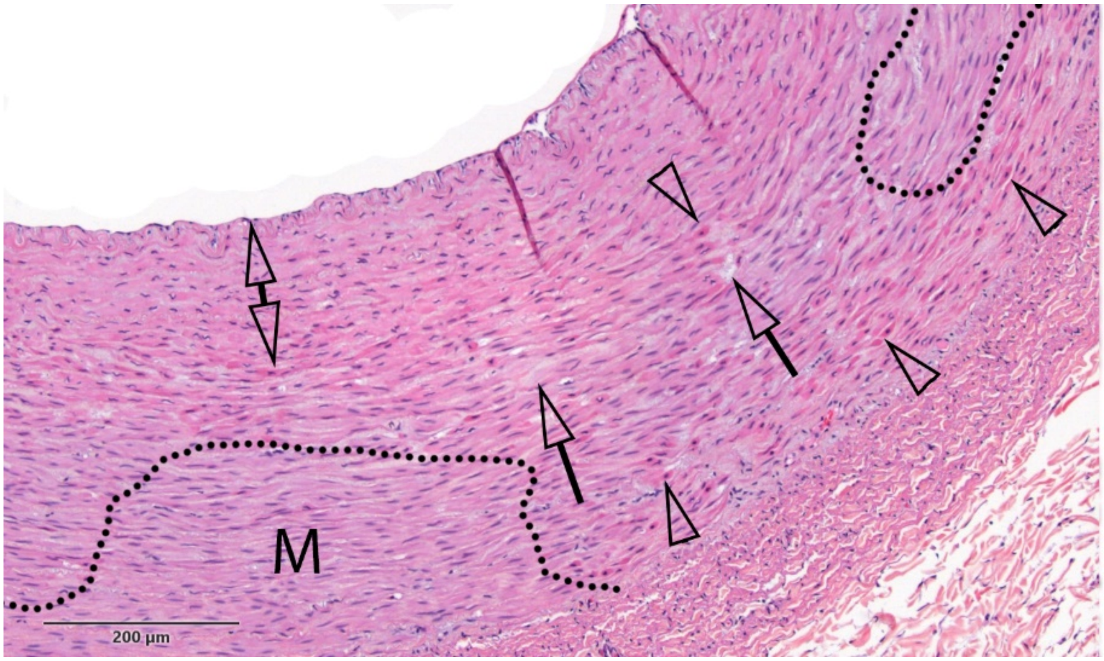


**Figure 23.** Segment 3L (H&E). C5 (1720 nm – 50 J/cm$^2$). Black dotted lines = boundary between the intact outer and mid media (M) and the affected media showing hypereosinophilic and contracted smooth muscle cells (clear arrowheads) and increased interstitial clear spaces (clear arrows), the innermost media (clear double arrow) shows pyknotic nuclei and hypereosinophilic smooth muscle cells (typically suggestive of compressive injury).

## C6 (1720 nm - 200 J/cm$^2$)

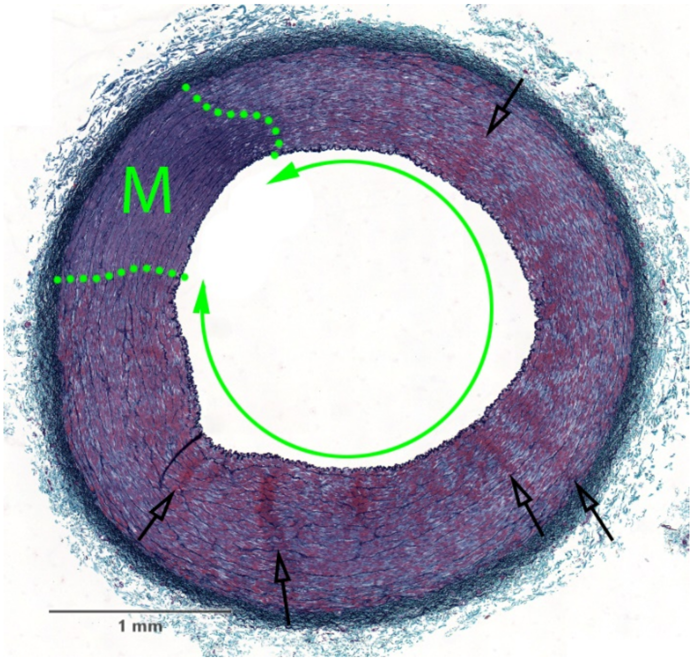


**Figure 24.** Segment 1P (GET). C6 (1720 nm – 200 J/cm$^2$). Section showing widespread media necrosis (circular green double arrow) and a short segment of intact media (M, delineated by green dotted line); clear arrows = radial clusters of hyperchromatic and shrunken smooth muscle cells evoking contraction bands.

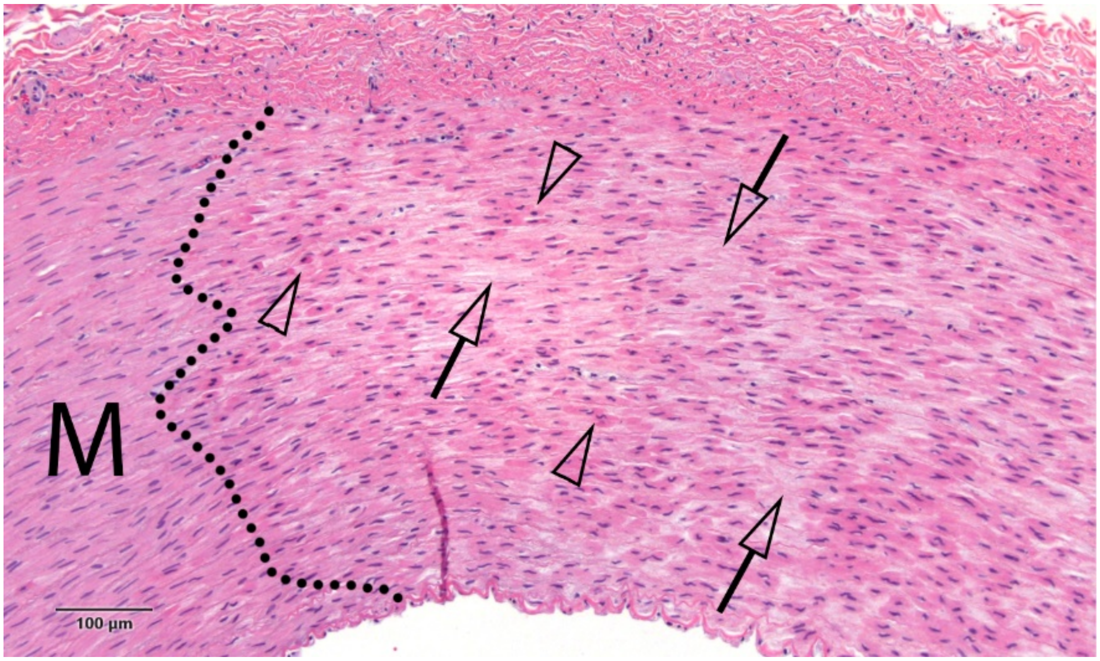


**Figure 25.** Segment 1P (H&E). C6 (1720 nm – 200 J/cm$^2$). Black dotted line = radial boundary between the intact media (M) and the affected media showing widespread hypereosinophilic and contracted smooth muscle cells (clear arrowheads) and increased interstitial clear spaces (clear arrows).

## C6 (1720 nm - 200 J/cm$^2$) (CONTINUED)

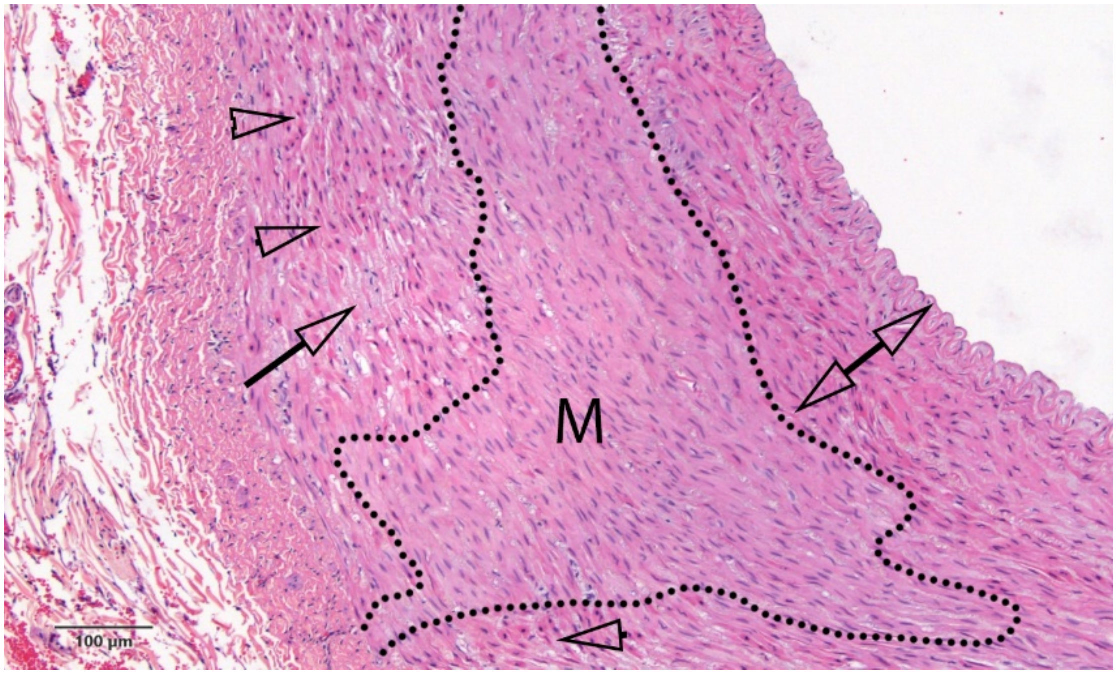


**Figure 26.** Segment 3O (H&E). C6 (1720 nm – 200 J/cm$^2$). Black dotted line = boundary between the intact outer and mid media (M) and the affected media showing widespread hypereosinophilic and contracted smooth muscle cells (clear arrowheads) and increased interstitial clear spaces (clear arrows); the innermost media (clear double arrow) shows pyknotic nuclei and hypereosinophilic smooth muscle cells (typically suggestive of compressive injury).

## Conclusion

Changes induced by treatment overlap with changes induced by catheterization +/- vasospasm (*i.e.*, compressive necrosis and stretch necrosis). Outer media necrosis characterized by smooth muscle cell hypereosinophilia and contraction is most characteristic. Treatment effect was clear in C3 (1064 nm; 700 J/cm$^2$) and C5 < C6 (1720 nm; 50, 200 J/cm$^2$, respectively). Possible treatment effects sporadically at lower doses (C1, C2 and C4). The tissue damage at these levels are not considered life threatening and are expected to heal. While nothing in this study represents a safety concern, a chronic time period (approximately 4 weeks) would be required to confirm that healing is uneventful.

Serge D. Rousselle, DVM, DACVP
Study Pathologist

24 MAR 21
Date

# Appendix A. Tabulated Microscopic Data

| HJC19-339 | | INJURY | | | | | | | | | INFLAMMATION | | | | | | | | | |
|---|---|---|---|---|---|---|---|---|---|---|---|---|---|---|---|---|---|---|---|---|
| | | Vessel Wall Injury | | Severe Injury (Disruptions) | | | Thermal Injury | | | | See pathology narrative for scoring definitions. | | | | | | | | | |
| | | | | | | | | | | | Mural Inflammation | | *Inflammatory Cell Types* | | | | | Inflammation Distribution | | |
| | | Injury Mean | Injury Median | Mural Erosion | Mural Dissection | Mural Avulsion | Mural Acute Thermnal Injury | Media Necrosis | Media Hyalinization | Hyalinized Collagen without Other Thermal Injury | Inflammation Mean | Inflammation Median | *Neutrophils* | *Eosinophils* | *Lymphocytes* | *Histiocytes* | *Foreign Body Giant cells* | Inflammation, Neointima | Inflammation, Media | Inflammation, Adventitia |
| C0 | n | 9 | 9 | 9 | 9 | 9 | 9 | 9 | 9 | 9 | 9 | 9 | 9 | 9 | 9 | 9 | 9 | 9 | 9 | 9 |
| | Mean | 0.0 | 0.0 | 0.0 | 0.0 | 0.0 | 0.0 | 0.7 | 0.0 | 0.3 | 0.1 | 0.0 | 0.4 | 0.0 | 0.0 | 0.0 | 0.0 | 0.0 | 0.0 | 0.4 |
| | *StDev* | 0.0 | 0.0 | 0.0 | 0.0 | 0.0 | 0.0 | 0.7 | 0.0 | 0.7 | 0.2 | 0.2 | 0.5 | 0.0 | 0.0 | 0.0 | 0.0 | 0.0 | 0.0 | 0.5 |
| C1 | n | 9 | 9 | 9 | 9 | 9 | 9 | 9 | 9 | 9 | 9 | 9 | 9 | 9 | 9 | 9 | 9 | 9 | 9 | 9 |
| | Mean | 0.0 | 0.0 | 0.0 | 0.0 | 0.0 | 0.0 | 1.1 | 0.0 | 0.1 | 0.2 | 0.0 | 0.7 | 0.0 | 0.0 | 0.0 | 0.0 | 0.0 | 0.0 | 0.7 |
| | *StDev* | 0.0 | 0.0 | 0.0 | 0.0 | 0.0 | 0.0 | 0.6 | 0.0 | 0.3 | 0.2 | 0.2 | 0.5 | 0.0 | 0.0 | 0.0 | 0.0 | 0.0 | 0.0 | 0.5 |
| C2 | n | 7 | 7 | 7 | 7 | 7 | 7 | 7 | 7 | 7 | 7 | 7 | 7 | 7 | 7 | 7 | 7 | 7 | 7 | 7 |
| | Mean | 0.0 | 0.0 | 0.0 | 0.0 | 0.0 | 0.0 | 0.7 | 0.0 | 0.0 | 0.1 | 0.0 | 0.1 | 0.0 | 0.0 | 0.0 | 0.0 | 0.0 | 0.0 | 0.1 |
| | *StDev* | 0.0 | 0.0 | 0.0 | 0.0 | 0.0 | 0.0 | 1.0 | 0.0 | 0.0 | 0.2 | 0.2 | 0.4 | 0.0 | 0.0 | 0.0 | 0.0 | 0.0 | 0.0 | 0.4 |
| C3 | n | 5 | 5 | 5 | 5 | 5 | 5 | 5 | 5 | 5 | 5 | 5 | 5 | 5 | 5 | 5 | 5 | 5 | 5 | 5 |
| | Mean | 0.0 | 0.0 | 0.0 | 0.0 | 0.0 | 0.0 | 1.4 | 0.0 | 0.0 | 0.2 | 0.0 | 0.6 | 0.0 | 0.0 | 0.0 | 0.0 | 0.0 | 0.0 | 0.6 |
| | *StDev* | 0.0 | 0.0 | 0.0 | 0.0 | 0.0 | 0.0 | 0.5 | 0.0 | 0.0 | 0.2 | 0.2 | 0.5 | 0.0 | 0.0 | 0.0 | 0.0 | 0.0 | 0.0 | 0.5 |
| C4 | n | 4 | 4 | 4 | 4 | 4 | 4 | 4 | 4 | 4 | 4 | 4 | 4 | 4 | 4 | 4 | 4 | 4 | 4 | 4 |
| | Mean | 0.0 | 0.0 | 0.0 | 0.0 | 0.0 | 0.0 | 0.5 | 0.0 | 0.5 | 0.4 | 0.5 | 0.8 | 0.0 | 0.0 | 0.0 | 0.0 | 0.0 | 0.0 | 0.8 |
| | *StDev* | 0.0 | 0.0 | 0.0 | 0.0 | 0.0 | 0.0 | 1.0 | 0.0 | 1.0 | 0.3 | 0.3 | 0.5 | 0.0 | 0.0 | 0.0 | 0.0 | 0.0 | 0.0 | 0.5 |
| C5 | n | 10 | 10 | 10 | 10 | 10 | 10 | 10 | 10 | 10 | 10 | 10 | 10 | 10 | 10 | 10 | 10 | 10 | 10 | 10 |
| | Mean | 0.0 | 0.0 | 0.0 | 0.0 | 0.0 | 0.2 | 2.2 | 0.0 | 0.0 | 0.2 | 0.2 | 0.6 | 0.0 | 0.0 | 0.0 | 0.0 | 0.0 | 0.0 | 0.6 |
| | *StDev* | 0.0 | 0.0 | 0.0 | 0.0 | 0.0 | 0.4 | 0.8 | 0.0 | 0.0 | 0.2 | 0.2 | 0.5 | 0.0 | 0.0 | 0.0 | 0.0 | 0.0 | 0.0 | 0.5 |
| C6 | n | 4 | 4 | 4 | 4 | 4 | 4 | 4 | 4 | 4 | 4 | 4 | 4 | 4 | 4 | 4 | 4 | 4 | 4 | 4 |
| | Mean | 0.0 | 0.0 | 0.0 | 0.0 | 0.0 | 0.0 | 3.3 | 0.0 | 0.0 | 0.0 | 0.0 | 0.0 | 0.0 | 0.0 | 0.0 | 0.0 | 0.0 | 0.0 | 0.0 |
| | *StDev* | 0.0 | 0.0 | 0.0 | 0.0 | 0.0 | 0.0 | 0.5 | 0.0 | 0.0 | 0.0 | 0.0 | 0.0 | 0.0 | 0.0 | 0.0 | 0.0 | 0.0 | 0.0 | 0.0 |

n = Number of segments used in calculations;
StDev = Standard Deviation

| HJC19-339 | | HEALING | | | | | | | | | | | | | | | | | | |
|---|---|---|---|---|---|---|---|---|---|---|---|---|---|---|---|---|---|---|---|---|
| | | See pathology narrative for scoring definitions. | | | | | | | | | | | | | | | | | | |
| | | Endovascular | | | | | | | | Vessel Wall | | | | | | | | Other | | |
| | | Endothelialization | Endothelium Erosion (terminal or artifact) | Leukocyte Margination | Thrombosis | Occlusion | Neointima Fibrin | Neointima Maturity | Neointima - Hyalinization | Hemosiderin/Hemorrhage, Vessel Wall | Mural - Necrosis/Hyalinization | Mural Calcification | Mural Neovascularization | Media Fibrosis | Adventitia Edema | Adventitial Fibrosis | Adventitia Granulation Tissue | Vasculitis/Perivasculitis- Branch | Branch - Thrombus | Aneurysmal Dilation |
| C0 | n | 9 | 9 | 9 | 9 | 9 | 9 | 9 | 9 | 9 | 9 | 9 | 9 | 9 | 9 | 9 | 9 | 9 | 9 | 9 |
| | Mean | **4.0** | **1.8** | 0.0 | 0.0 | 0.0 | 0.0 | 0.0 | 0.0 | **0.8** | 0.0 | 0.0 | 0.0 | 0.0 | **0.1** | 0.0 | 0.0 | 0.0 | 0.0 | 0.0 |
| | *StDev* | *0.0* | **1.1** | *0.0* | *0.0* | *0.0* | *0.0* | *0.0* | *0.0* | **0.7** | *0.0* | *0.0* | *0.0* | *0.0* | **0.3** | *0.0* | *0.0* | *0.0* | *0.0* | *0.0* |
| C1 | n | 9 | 9 | 9 | 9 | 9 | 9 | 9 | 9 | 9 | 9 | 9 | 9 | 9 | 9 | 9 | 9 | 9 | 9 | 9 |
| | Mean | **4.0** | **1.3** | 0.0 | 0.0 | 0.0 | 0.0 | 0.0 | 0.0 | **0.9** | 0.0 | 0.0 | 0.0 | 0.0 | **0.6** | 0.0 | 0.0 | 0.0 | 0.0 | 0.0 |
| | *StDev* | *0.0* | **0.5** | *0.0* | *0.0* | *0.0* | *0.0* | *0.0* | *0.0* | **0.3** | *0.0* | *0.0* | *0.0* | *0.0* | **0.7** | *0.0* | *0.0* | *0.0* | *0.0* | *0.0* |
| C2 | n | 7 | 7 | 7 | 7 | 7 | 7 | 7 | 7 | 7 | 7 | 7 | 7 | 7 | 7 | 7 | 7 | 7 | 7 | 7 |
| | Mean | **4.0** | **0.4** | 0.0 | 0.0 | 0.0 | 0.0 | 0.0 | 0.0 | **0.7** | 0.0 | 0.0 | 0.0 | 0.0 | 0.0 | 0.0 | 0.0 | 0.0 | 0.0 | 0.0 |
| | *StDev* | *0.0* | **0.5** | *0.0* | *0.0* | *0.0* | *0.0* | *0.0* | *0.0* | **0.5** | *0.0* | *0.0* | *0.0* | *0.0* | *0.0* | *0.0* | *0.0* | *0.0* | *0.0* | *0.0* |
| C3 | n | 5 | 5 | 5 | 5 | 5 | 5 | 5 | 5 | 5 | 5 | 5 | 5 | 5 | 5 | 5 | 5 | 5 | 5 | 5 |
| | Mean | **4.0** | **1.2** | 0.0 | 0.0 | 0.0 | 0.0 | 0.0 | 0.0 | **1.0** | 0.0 | 0.0 | 0.0 | 0.0 | 0.0 | 0.0 | 0.0 | 0.0 | 0.0 | 0.0 |
| | *StDev* | *0.0* | **0.4** | *0.0* | *0.0* | *0.0* | *0.0* | *0.0* | *0.0* | *0.0* | *0.0* | *0.0* | *0.0* | *0.0* | *0.0* | *0.0* | *0.0* | *0.0* | *0.0* | *0.0* |
| C4 | n | 4 | 4 | 4 | 4 | 4 | 4 | 4 | 4 | 4 | 4 | 4 | 4 | 4 | 4 | 4 | 4 | 4 | 4 | 4 |
| | Mean | **4.0** | **0.8** | 0.0 | 0.0 | 0.0 | 0.0 | 0.0 | 0.0 | **0.3** | 0.0 | 0.0 | 0.0 | 0.0 | 0.0 | 0.0 | 0.0 | 0.0 | 0.0 | 0.0 |
| | *StDev* | *0.0* | **0.5** | *0.0* | *0.0* | *0.0* | *0.0* | *0.0* | *0.0* | **0.5** | *0.0* | *0.0* | *0.0* | *0.0* | *0.0* | *0.0* | *0.0* | *0.0* | *0.0* | *0.0* |
| C5 | n | 10 | 10 | 10 | 10 | 10 | 10 | 10 | 10 | 10 | 10 | 10 | 10 | 10 | 10 | 10 | 10 | 10 | 10 | 10 |
| | Mean | **4.0** | **1.9** | 0.0 | 0.0 | 0.0 | 0.0 | 0.0 | 0.0 | **0.6** | 0.0 | 0.0 | 0.0 | 0.0 | 0.0 | 0.0 | 0.0 | 0.0 | 0.0 | 0.0 |
| | *StDev* | *0.0* | **0.9** | *0.0* | *0.0* | *0.0* | *0.0* | *0.0* | *0.0* | **0.5** | *0.0* | *0.0* | *0.0* | *0.0* | *0.0* | *0.0* | *0.0* | *0.0* | *0.0* | *0.0* |
| C6 | n | 4 | 4 | 4 | 4 | 4 | 4 | 4 | 4 | 4 | 4 | 4 | 4 | 4 | 4 | 4 | 4 | 4 | 4 | 4 |
| | Mean | **4.0** | **3.5** | 0.0 | 0.0 | 0.0 | 0.0 | 0.0 | 0.0 | **1.0** | 0.0 | 0.0 | 0.0 | 0.0 | 0.0 | 0.0 | 0.0 | 0.0 | 0.0 | 0.0 |
| | *StDev* | *0.0* | **0.6** | *0.0* | *0.0* | *0.0* | *0.0* | *0.0* | *0.0* | **0.8** | *0.0* | *0.0* | *0.0* | *0.0* | *0.0* | *0.0* | *0.0* | *0.0* | *0.0* | *0.0* |

n = Number of segments used in calculations;
StDev = Standard Deviation

| HJC19-339 | | | | | INJURY | | | | | | | | | | | | | INFLAMMATION | | | | | | | | | | | | | | |
|---|---|---|---|---|---|---|---|---|---|---|---|---|---|---|---|---|---|---|---|---|---|---|---|---|---|---|---|---|---|---|---|
| | | | | | Vessel Wall Injury (scored per quadrant) | | | | | | Severe Injury (Disruptions) | | | Thermal Injury | | | | See pathology narrative for scoring definitions. Mural Inflammation (scored per quadrant) | | | | | | *Inflammatory Cell Types* | | | | | Inflammation Distribution | | |
| Wavelength (nm) | Condition Number | Fluence (J/cm$^2$) | Specimen (Artery) | Segment | I | II | III | IV | Injury Mean | Injury Median | Mural Erosion | Mural Dissection | Mural Avulsion | Mural Acute Thermal Injury | Media Necrosis | Media Hyalinization | Hyalinized Collagen without Other Thermal Injury | I | II | III | IV | Inflammation Mean | Inflammation Median | *Neutrophils* | *Eosinophils* | *Lymphocytes* | *Histiocytes* | *Foreign Body Giant cells* | Inflammation, Neointima | Inflammation, Media | Inflammation, Adventitia |
| None | C0 | 0 | 1 | 1-A | 0 | 0 | 0 | 0 | 0.0 | 0.0 | 0 | 0 | 0 | 0 | **1** | 0 | 0 | 0 | **1** | 0 | 0 | **0.3** | 0.0 | **1** | 0 | 0 | 0 | 0 | 0 | 0 | **1** |
| | | | | 1-B | 0 | 0 | 0 | 0 | 0.0 | 0.0 | 0 | 0 | 0 | 0 | **1** | 0 | 0 | 0 | 0 | 0 | 0 | 0.0 | 0.0 | 0 | 0 | 0 | 0 | 0 | 0 | 0 | 0 |
| | | | | 1-C | 0 | 0 | 0 | 0 | 0.0 | 0.0 | 0 | 0 | 0 | 0 | **2** | 0 | 0 | 0 | **1** | 0 | 0 | **0.3** | 0.0 | **1** | 0 | 0 | 0 | 0 | 0 | 0 | **1** |
| | | | | 1-D** | 0 | 0 | 0 | 0 | 0.0 | 0.0 | 0 | 0 | 0 | 0 | **1** | 0 | 0 | 0 | 0 | 0 | **1** | **0.3** | 0.0 | **1** | 0 | 0 | 0 | 0 | 0 | 0 | **1** |
| | | | 2 | 2-A | 0 | 0 | 0 | 0 | 0.0 | 0.0 | 0 | 0 | 0 | 0 | **1** | 0 | **1** | 0 | 0 | 0 | 0 | 0.0 | 0.0 | 0 | 0 | 0 | 0 | 0 | 0 | 0 | 0 |
| | | | | 2-B | 0 | 0 | 0 | 0 | 0.0 | 0.0 | 0 | 0 | 0 | 0 | 0 | 0 | 0 | 0 | 0 | 0 | 0 | 0.0 | 0.0 | 0 | 0 | 0 | 0 | 0 | 0 | 0 | 0 |
| | | | | 2-P*** | 0 | 0 | 0 | 0 | 0.0 | 0.0 | 0 | 0 | 0 | 0 | 0 | 0 | 0 | 0 | 0 | 0 | 0 | 0.0 | 0.0 | 0 | 0 | 0 | 0 | 0 | 0 | 0 | 0 |
| | | | 3 | 3-A | 0 | 0 | 0 | 0 | 0.0 | 0.0 | 0 | 0 | 0 | 0 | 0 | 0 | 0 | 0 | 0 | 0 | 0 | 0.0 | 0.0 | 0 | 0 | 0 | 0 | 0 | 0 | 0 | 0 |
| | | | | 3-B | 0 | 0 | 0 | 0 | 0.0 | 0.0 | 0 | 0 | 0 | 0 | 0 | 0 | **2** | **1** | **1** | 0 | 0 | **0.5** | **0.5** | **1** | 0 | 0 | 0 | 0 | 0 | 0 | **1** |

* = Treatment applied to ~1/4 of circumference;
** = Section is incomplete;
*** = Marker suture hole present

| HJC19-339 | | | | | INJURY | | | | | | | | | | | | | INFLAMMATION | | | | | | | | | | | | | |
|---|---|---|---|---|---|---|---|---|---|---|---|---|---|---|---|---|---|---|---|---|---|---|---|---|---|---|---|---|---|---|---|
| | | | | | Vessel Wall Injury (scored per quadrant) | | | | | | Severe Injury (Disruptions) | | | Thermal Injury | | | | See pathology narrative for scoring definitions. | | | | | | | | | | | | | |
| | | | | | | | | | | | | | | | | | | Mural Inflammation (scored per quadrant) | | | | | | *Inflammatory Cell Types* | | | | | Inflammation Distribution | | |
| Wavelength (nm) | Condition Number | Fluence (J/cm$^2$) | Specimen (Artery) | Segment | I | II | III | IV | Injury Mean | Injury Median | Mural Erosion | Mural Dissection | Mural Avulsion | Mural Acute Thermal Injury | Media Necrosis | Media Hyalinization | Hyalinized Collagen without Other Thermal Injury | I | II | III | IV | Inflammation Mean | Inflammation Median | *Neutrophils* | *Eosinophils* | *Lymphocytes* | *Histiocytes* | *Foreign Body Giant cells* | Inflammation, Neointima | Inflammation, Media | Inflammation, Adventitia |
| 1064 | C1 | 8.3 | 1 | 1-E* | 0 | 0 | 0 | 0 | 0.0 | 0.0 | 0 | 0 | 0 | 0 | 1 | 0 | 0 | 1 | 0 | 0 | 0 | 0.3 | 0.0 | 1 | 0 | 0 | 0 | 0 | 0 | 0 | 1 |
| | | | | 1-F* | 0 | 0 | 0 | 0 | 0.0 | 0.0 | 0 | 0 | 0 | 0 | 2 | 0 | 0 | 0 | 0 | 0 | 0 | 0.0 | 0.0 | 0 | 0 | 0 | 0 | 0 | 0 | 0 | 0 |
| | | | | 1-G | 0 | 0 | 0 | 0 | 0.0 | 0.0 | 0 | 0 | 0 | 0 | 1 | 0 | 0 | 0 | 0 | 0 | 1 | 0.3 | 0.0 | 1 | 0 | 0 | 0 | 0 | 0 | 0 | 1 |
| | | | | 1-H | 0 | 0 | 0 | 0 | 0.0 | 0.0 | 0 | 0 | 0 | 0 | 2 | 0 | 0 | 0 | 0 | 1 | 1 | 0.5 | 0.5 | 1 | 0 | 0 | 0 | 0 | 0 | 0 | 1 |
| | | | 2 | 2-C | 0 | 0 | 0 | 0 | 0.0 | 0.0 | 0 | 0 | 0 | 0 | 1 | 0 | 0 | 0 | 1 | 0 | 0 | 0.3 | 0.0 | 1 | 0 | 0 | 0 | 0 | 0 | 0 | 1 |
| | | | | 2-D | 0 | 0 | 0 | 0 | 0.0 | 0.0 | 0 | 0 | 0 | 0 | 1 | 0 | 1 | 0 | 0 | 0 | 0 | 0.0 | 0.0 | 0 | 0 | 0 | 0 | 0 | 0 | 0 | 0 |
| | | | | 2-E | 0 | 0 | 0 | 0 | 0.0 | 0.0 | 0 | 0 | 0 | 0 | 1 | 0 | 0 | 0 | 0 | 0 | 1 | 0.3 | 0.0 | 1 | 0 | 0 | 0 | 0 | 0 | 0 | 1 |
| | | | | 2-F | 0 | 0 | 0 | 0 | 0.0 | 0.0 | 0 | 0 | 0 | 0 | 1 | 0 | 0 | 0 | 0 | 0 | 0 | 0.0 | 0.0 | 0 | 0 | 0 | 0 | 0 | 0 | 0 | 0 |
| | | | | 2-G | 0 | 0 | 0 | 0 | 0.0 | 0.0 | 0 | 0 | 0 | 0 | 0 | 0 | 0 | 0 | 0 | 1 | 1 | 0.5 | 0.5 | 1 | 0 | 0 | 0 | 0 | 0 | 0 | 1 |
| | C2 | 100 | 1 | 1-I | 0 | 0 | 0 | 0 | 0.0 | 0.0 | 0 | 0 | 0 | 0 | 2 | 0 | 0 | 1 | 1 | 0 | 0 | 0.5 | 0.5 | 1 | 0 | 0 | 0 | 0 | 0 | 0 | 1 |
| | | | | 1-J | 0 | 0 | 0 | 0 | 0.0 | 0.0 | 0 | 0 | 0 | 0 | 2 | 0 | 0 | 0 | 0 | 0 | 0 | 0.0 | 0.0 | 0 | 0 | 0 | 0 | 0 | 0 | 0 | 0 |
| | | | 2 | 2-H | 0 | 0 | 0 | 0 | 0.0 | 0.0 | 0 | 0 | 0 | 0 | 0 | 0 | 0 | 0 | 0 | 0 | 0 | 0.0 | 0.0 | 0 | 0 | 0 | 0 | 0 | 0 | 0 | 0 |
| | | | | 2-I | 0 | 0 | 0 | 0 | 0.0 | 0.0 | 0 | 0 | 0 | 0 | 0 | 0 | 0 | 0 | 0 | 0 | 0 | 0.0 | 0.0 | 0 | 0 | 0 | 0 | 0 | 0 | 0 | 0 |
| | | | | 2-J | 0 | 0 | 0 | 0 | 0.0 | 0.0 | 0 | 0 | 0 | 0 | 0 | 0 | 0 | 0 | 0 | 0 | 0 | 0.0 | 0.0 | 0 | 0 | 0 | 0 | 0 | 0 | 0 | 0 |
| | | | | 2-K | 0 | 0 | 0 | 0 | 0.0 | 0.0 | 0 | 0 | 0 | 0 | 0 | 0 | 0 | 0 | 0 | 0 | 0 | 0.0 | 0.0 | 0 | 0 | 0 | 0 | 0 | 0 | 0 | 0 |
| | | | | 2-L | 0 | 0 | 0 | 0 | 0.0 | 0.0 | 0 | 0 | 0 | 0 | 1 | 0 | 0 | 0 | 0 | 0 | 0 | 0.0 | 0.0 | 0 | 0 | 0 | 0 | 0 | 0 | 0 | 0 |
| | C3 | 700 | 1 | 1-K | 0 | 0 | 0 | 0 | 0.0 | 0.0 | 0 | 0 | 0 | 0 | 2 | 0 | 0 | 1 | 0 | 0 | 1 | 0.5 | 0.5 | 1 | 0 | 0 | 0 | 0 | 0 | 0 | 1 |
| | | | | 1-L | 0 | 0 | 0 | 0 | 0.0 | 0.0 | 0 | 0 | 0 | 0 | 2 | 0 | 0 | 0 | 1 | 0 | 0 | 0.3 | 0.0 | 1 | 0 | 0 | 0 | 0 | 0 | 0 | 1 |
| | | | 2 | 2-M | 0 | 0 | 0 | 0 | 0.0 | 0.0 | 0 | 0 | 0 | 0 | 1 | 0 | 0 | 0 | 0 | 0 | 1 | 0.3 | 0.0 | 1 | 0 | 0 | 0 | 0 | 0 | 0 | 1 |
| | | | | 2-N | 0 | 0 | 0 | 0 | 0.0 | 0.0 | 0 | 0 | 0 | 0 | 1 | 0 | 0 | 0 | 0 | 0 | 0 | 0.0 | 0.0 | 0 | 0 | 0 | 0 | 0 | 0 | 0 | 0 |
| | | | | 2-O | 0 | 0 | 0 | 0 | 0.0 | 0.0 | 0 | 0 | 0 | 0 | 1 | 0 | 0 | 0 | 0 | 0 | 0 | 0.0 | 0.0 | 0 | 0 | 0 | 0 | 0 | 0 | 0 | 0 |

* = Treatment applied to ~1/4 of circumference;
** = Section is incomplete;
*** = Marker suture hole present

| HJC19-339 | | | | | INJURY | | | | | | | | | | | | | INFLAMMATION | | | | | | | | | | | | | |
|---|---|---|---|---|---|---|---|---|---|---|---|---|---|---|---|---|---|---|---|---|---|---|---|---|---|---|---|---|---|---|---|
| | | | | | Vessel Wall Injury (scored per quadrant) | | | | | | Severe Injury (Disruptions) | | | Thermal Injury | | | | See pathology narrative for scoring definitions. | | | | | | | | | | | | | |
| | | | | | | | | | | | | | | | | | | Mural Inflammation (scored per quadrant) | | | | | | *Inflammatory Cell Types* | | | | | Inflammation Distribution | | |
| Wavelength (nm) | Condition Number | Fluence (J/cm$^2$) | Specimen (Artery) | Segment | I | II | III | IV | Injury Mean | Injury Median | Mural Erosion | Mural Dissection | Mural Avulsion | Mural Acute Thermal Injury | Media Necrosis | Media Hyalinization | Hyalinized Collagen without Other Thermal Injury | I | II | III | IV | Inflammation Mean | Inflammation Median | *Neutrophils* | *Eosinophils* | *Lymphocytes* | *Histiocytes* | *Foreign Body Giant cells* | Inflammation, Neointima | Inflammation, Media | Inflammation, Adventitia |
| 1720 | C4 | 8.3 | 1 | 1-M | 0 | 0 | 0 | 0 | 0.0 | 0.0 | 0 | 0 | 0 | 0 | 2 | 0 | 0 | 1 | 0 | 0 | 1 | 0.5 | 0.5 | 1 | 0 | 0 | 0 | 0 | 0 | 0 | 1 |
| | | | 3 | 3-C | 0 | 0 | 0 | 0 | 0.0 | 0.0 | 0 | 0 | 0 | 0 | 0 | 0 | 2 | 1 | 0 | 0 | 1 | 0.5 | 0.5 | 1 | 0 | 0 | 0 | 0 | 0 | 0 | 1 |
| | | | | 3-D | 0 | 0 | 0 | 0 | 0.0 | 0.0 | 0 | 0 | 0 | 0 | 0 | 0 | 0 | 0 | 0 | 0 | 0 | 0.0 | 0.0 | 0 | 0 | 0 | 0 | 0 | 0 | 0 | 0 |
| | | | | 3-E | 0 | 0 | 0 | 0 | 0.0 | 0.0 | 0 | 0 | 0 | 0 | 0 | 0 | 0 | 1 | 0 | 0 | 1 | 0.5 | 0.5 | 1 | 0 | 0 | 0 | 0 | 0 | 0 | 1 |
| | C5 | 50 | 1 | 1-N | 0 | 0 | 0 | 0 | 0.0 | 0.0 | 0 | 0 | 0 | 1 | 2 | 0 | 0 | 0 | 1 | 0 | 0 | 0.3 | 0.0 | 1 | 0 | 0 | 0 | 0 | 0 | 0 | 1 |
| | | | | 1-O | 0 | 0 | 0 | 0 | 0.0 | 0.0 | 0 | 0 | 0 | 1 | 3 | 0 | 0 | 0 | 1 | 1 | 0 | 0.5 | 0.5 | 1 | 0 | 0 | 0 | 0 | 0 | 0 | 1 |
| | | | 3 | 3-F | 0 | 0 | 0 | 0 | 0.0 | 0.0 | 0 | 0 | 0 | 0 | 1 | 0 | 0 | 1 | 0 | 0 | 1 | 0.5 | 0.5 | 1 | 0 | 0 | 0 | 0 | 0 | 0 | 1 |
| | | | | 3-G | 0 | 0 | 0 | 0 | 0.0 | 0.0 | 0 | 0 | 0 | 0 | 1 | 0 | 0 | 0 | 0 | 1 | 0 | 0.3 | 0.0 | 1 | 0 | 0 | 0 | 0 | 0 | 0 | 1 |
| | | | | 3-H | 0 | 0 | 0 | 0 | 0.0 | 0.0 | 0 | 0 | 0 | 0 | 2 | 0 | 0 | 0 | 1 | 1 | 0 | 0.5 | 0.5 | 1 | 0 | 0 | 0 | 0 | 0 | 0 | 1 |
| | | | | 3-I | 0 | 0 | 0 | 0 | 0.0 | 0.0 | 0 | 0 | 0 | 0 | 2 | 0 | 0 | 0 | 1 | 0 | 0 | 0.3 | 0.0 | 1 | 0 | 0 | 0 | 0 | 0 | 0 | 1 |
| | | | | 3-J | 0 | 0 | 0 | 0 | 0.0 | 0.0 | 0 | 0 | 0 | 0 | 2 | 0 | 0 | 0 | 0 | 0 | 0 | 0.0 | 0.0 | 0 | 0 | 0 | 0 | 0 | 0 | 0 | 0 |
| | | | | 3-K | 0 | 0 | 0 | 0 | 0.0 | 0.0 | 0 | 0 | 0 | 0 | 3 | 0 | 0 | 0 | 0 | 0 | 0 | 0.0 | 0.0 | 0 | 0 | 0 | 0 | 0 | 0 | 0 | 0 |
| | | | | 3-L | 0 | 0 | 0 | 0 | 0.0 | 0.0 | 0 | 0 | 0 | 0 | 3 | 0 | 0 | 0 | 0 | 0 | 0 | 0.0 | 0.0 | 0 | 0 | 0 | 0 | 0 | 0 | 0 | 0 |
| | | | | 3-M | 0 | 0 | 0 | 0 | 0.0 | 0.0 | 0 | 0 | 0 | 0 | 3 | 0 | 0 | 0 | 0 | 0 | 0 | 0.0 | 0.0 | 0 | 0 | 0 | 0 | 0 | 0 | 0 | 0 |
| | C6 | 200 | 1 | 1-P | 0 | 0 | 0 | 0 | 0.0 | 0.0 | 0 | 0 | 0 | 0 | 3 | 0 | 0 | 0 | 0 | 0 | 0 | 0.0 | 0.0 | 0 | 0 | 0 | 0 | 0 | 0 | 0 | 0 |
| | | | 3 | 3-N | 0 | 0 | 0 | 0 | 0.0 | 0.0 | 0 | 0 | 0 | 0 | 4 | 0 | 0 | 0 | 0 | 0 | 0 | 0.0 | 0.0 | 0 | 0 | 0 | 0 | 0 | 0 | 0 | 0 |
| | | | | 3-O | 0 | 0 | 0 | 0 | 0.0 | 0.0 | 0 | 0 | 0 | 0 | 3 | 0 | 0 | 0 | 0 | 0 | 0 | 0.0 | 0.0 | 0 | 0 | 0 | 0 | 0 | 0 | 0 | 0 |
| | | | | 3-P | 0 | 0 | 0 | 0 | 0.0 | 0.0 | 0 | 0 | 0 | 0 | 3 | 0 | 0 | 0 | 0 | 0 | 0 | 0.0 | 0.0 | 0 | 0 | 0 | 0 | 0 | 0 | 0 | 0 |

* = Treatment applied to ~1/4 of circumference;
** = Section is incomplete;
*** = Marker suture hole present

| HJC19-339 | | | | | HEALING | | | | | | | | | | | | | | | | | | |
|---|---|---|---|---|---|---|---|---|---|---|---|---|---|---|---|---|---|---|---|---|---|---|---|
| | | | | | See pathology narrative for scoring definitions. | | | | | | | | | | | | | | | | | | |
| | | | | | Endovascular | | | | | | | | Vessel Wall | | | | | | | | Other | | |
| Wavelength (nm) | Condition Number | Fluence (J/cm$^2$) | Specimen (Artery) | Segment | Endothelialization | Endothelium Erosion (terminal or artifact) | Leukocyte Margination | Thrombosis | Occlusion | Neointima Fibrin | Neointima Maturity | Neointima - Hyalinization | Hemosiderin/Hemorrhage, Vessel Wall | Mural - Necrosis/Hyalinization | Mural Calcification | Mural Neovascularization | Media Fibrosis | Adventitia Edema | Adventitial Fibrosis | Adventitia Granulation Tissue | Vasculitis/Perivasculitis- Branch | Branch - Thrombus | Aneurysmal Dilation |
| None | C0 | 0 | 1 | 1-A | **4** | **1** | 0 | 0 | 0 | 0 | 0 | 0 | **1** | 0 | 0 | 0 | 0 | 0 | 0 | 0 | 0 | 0 | 0 |
| | | | | 1-B | **4** | **1** | 0 | 0 | 0 | 0 | 0 | 0 | **1** | 0 | 0 | 0 | 0 | 0 | 0 | 0 | 0 | 0 | 0 |
| | | | | 1-C | **4** | **1** | 0 | 0 | 0 | 0 | 0 | 0 | **1** | 0 | 0 | 0 | 0 | 0 | 0 | 0 | 0 | 0 | 0 |
| | | | | 1-D** | **4** | **2** | 0 | 0 | 0 | 0 | 0 | 0 | **1** | 0 | 0 | 0 | 0 | 0 | 0 | 0 | 0 | 0 | 0 |
| | | | 2 | 2-A | **4** | **4** | 0 | 0 | 0 | 0 | 0 | 0 | 0 | 0 | 0 | 0 | 0 | 0 | 0 | 0 | 0 | 0 | 0 |
| | | | | 2-B | **4** | **3** | 0 | 0 | 0 | 0 | 0 | 0 | **1** | 0 | 0 | 0 | 0 | **1** | 0 | 0 | 0 | 0 | 0 |
| | | | | 2-P*** | **4** | **2** | 0 | 0 | 0 | 0 | 0 | 0 | **2** | 0 | 0 | 0 | 0 | 0 | 0 | 0 | 0 | 0 | 0 |
| | | | 3 | 3-A | **4** | **1** | 0 | 0 | 0 | 0 | 0 | 0 | 0 | 0 | 0 | 0 | 0 | 0 | 0 | 0 | 0 | 0 | 0 |
| | | | | 3-B | **4** | **1** | 0 | 0 | 0 | 0 | 0 | 0 | 0 | 0 | 0 | 0 | 0 | 0 | 0 | 0 | 0 | 0 | 0 |

* = Treatment applied to ~1/4 of circumference;
** = Section is incomplete;
*** = Marker suture hole present

| HJC19-339 | | | | | HEALING | | | | | | | | | | | | | | | | | | | | |
|---|---|---|---|---|---|---|---|---|---|---|---|---|---|---|---|---|---|---|---|---|---|---|---|---|---|
| | | | | | See pathology narrative for scoring definitions. | | | | | | | | | | | | | | | | | | | | |
| | | | | | Endovascular | | | | | | | | Vessel Wall | | | | | | | | Other | | |
| Wavelength (nm) | Condition Number | Fluence (J/cm$^2$) | Specimen (Artery) | Segment | Endothelialization | Endothelium Erosion (terminal or artifact) | Leukocyte Margination | Thrombosis | Occlusion | Neointima Fibrin | Neointima Maturity | Neointima - Hyalinization | Hemosiderin/Hemorrhage, Vessel Wall | Mural - Necrosis/Hyalinization | Mural Calcification | Mural Neovascularization | Media Fibrosis | Adventitia Edema | Adventitial Fibrosis | Adventitia Granulation Tissue | Vasculitis/Perivasculitis- Branch | Branch - Thrombus | Aneurysmal Dilation |
| 1064 | C1 | 8.3 | 1 | 1-E* | 4 | 1 | 0 | 0 | 0 | 0 | 0 | 0 | 1 | 0 | 0 | 0 | 0 | 0 | 0 | 0 | 0 | 0 | 0 |
| | | | | 1-F* | 4 | 2 | 0 | 0 | 0 | 0 | 0 | 0 | 0 | 0 | 0 | 0 | 0 | 0 | 0 | 0 | 0 | 0 | 0 |
| | | | | 1-G | 4 | 1 | 0 | 0 | 0 | 0 | 0 | 0 | 1 | 0 | 0 | 0 | 0 | 0 | 0 | 0 | 0 | 0 | 0 |
| | | | | 1-H | 4 | 1 | 0 | 0 | 0 | 0 | 0 | 0 | 1 | 0 | 0 | 0 | 0 | 0 | 0 | 0 | 0 | 0 | 0 |
| | | | 2 | 2-C | 4 | 2 | 0 | 0 | 0 | 0 | 0 | 0 | 1 | 0 | 0 | 0 | 0 | 1 | 0 | 0 | 0 | 0 | 0 |
| | | | | 2-D | 4 | 2 | 0 | 0 | 0 | 0 | 0 | 0 | 1 | 0 | 0 | 0 | 0 | 0 | 0 | 0 | 0 | 0 | 0 |
| | | | | 2-E | 4 | 1 | 0 | 0 | 0 | 0 | 0 | 0 | 1 | 0 | 0 | 0 | 0 | 1 | 0 | 0 | 0 | 0 | 0 |
| | | | | 2-F | 4 | 1 | 0 | 0 | 0 | 0 | 0 | 0 | 1 | 0 | 0 | 0 | 0 | 2 | 0 | 0 | 0 | 0 | 0 |
| | | | | 2-G | 4 | 1 | 0 | 0 | 0 | 0 | 0 | 0 | 1 | 0 | 0 | 0 | 0 | 1 | 0 | 0 | 0 | 0 | 0 |
| | C2 | 100 | 1 | 1-I | 4 | 1 | 0 | 0 | 0 | 0 | 0 | 0 | 0 | 0 | 0 | 0 | 0 | 0 | 0 | 0 | 0 | 0 | 0 |
| | | | | 1-J | 4 | 1 | 0 | 0 | 0 | 0 | 0 | 0 | 0 | 0 | 0 | 0 | 0 | 0 | 0 | 0 | 0 | 0 | 0 |
| | | | 2 | 2-H | 4 | 0 | 0 | 0 | 0 | 0 | 0 | 0 | 1 | 0 | 0 | 0 | 0 | 0 | 0 | 0 | 0 | 0 | 0 |
| | | | | 2-I | 4 | 0 | 0 | 0 | 0 | 0 | 0 | 0 | 1 | 0 | 0 | 0 | 0 | 0 | 0 | 0 | 0 | 0 | 0 |
| | | | | 2-J | 4 | 0 | 0 | 0 | 0 | 0 | 0 | 0 | 1 | 0 | 0 | 0 | 0 | 0 | 0 | 0 | 0 | 0 | 0 |
| | | | | 2-K | 4 | 0 | 0 | 0 | 0 | 0 | 0 | 0 | 1 | 0 | 0 | 0 | 0 | 0 | 0 | 0 | 0 | 0 | 0 |
| | | | | 2-L | 4 | 1 | 0 | 0 | 0 | 0 | 0 | 0 | 1 | 0 | 0 | 0 | 0 | 0 | 0 | 0 | 0 | 0 | 0 |
| | C3 | 700 | 1 | 1-K | 4 | 1 | 0 | 0 | 0 | 0 | 0 | 0 | 1 | 0 | 0 | 0 | 0 | 0 | 0 | 0 | 0 | 0 | 0 |
| | | | | 1-L | 4 | 1 | 0 | 0 | 0 | 0 | 0 | 0 | 1 | 0 | 0 | 0 | 0 | 0 | 0 | 0 | 0 | 0 | 0 |
| | | | 2 | 2-M | 4 | 1 | 0 | 0 | 0 | 0 | 0 | 0 | 1 | 0 | 0 | 0 | 0 | 0 | 0 | 0 | 0 | 0 | 0 |
| | | | | 2-N | 4 | 1 | 0 | 0 | 0 | 0 | 0 | 0 | 1 | 0 | 0 | 0 | 0 | 0 | 0 | 0 | 0 | 0 | 0 |
| | | | | 2-O | 4 | 2 | 0 | 0 | 0 | 0 | 0 | 0 | 1 | 0 | 0 | 0 | 0 | 0 | 0 | 0 | 0 | 0 | 0 |

* = Treatment applied to ~1/4 of circumference;
** = Section is incomplete;
*** = Marker suture hole present

| HJC19-339 | | | | | HEALING | | | | | | | | | | | | | | | | | | |
|---|---|---|---|---|---|---|---|---|---|---|---|---|---|---|---|---|---|---|---|---|---|---|---|
| | | | | | See pathology narrative for scoring definitions. | | | | | | | | | | | | | | | | | | |
| | | | | | Endovascular | | | | | | | | Vessel Wall | | | | | | | | Other | | |
| Wavelength (nm) | Condition Number | Fluence ($J/cm^2$) | Specimen (Artery) | Segment | Endothelialization | Endothelium Erosion (terminal or artifact) | Leukocyte Margination | Thrombosis | Occlusion | Neointima Fibrin | Neointima Maturity | Neointima - Hyalinization | Hemosiderin/Hemorrhage, Vessel Wall | Mural - Necrosis/Hyalinization | Mural Calcification | Mural Neovascularization | Media Fibrosis | Adventitia Edema | Adventitial Fibrosis | Adventitia Granulation Tissue | Vasculitis/Perivasculitis- Branch | Branch - Thrombus | Aneurysmal Dilation |
| 1720 | C4 | 8.3 | 1 | 1-M | **4** | **1** | 0 | 0 | 0 | 0 | 0 | 0 | **1** | 0 | 0 | 0 | 0 | 0 | 0 | 0 | 0 | 0 | 0 |
| | | | 3 | 3-C | **4** | 0 | 0 | 0 | 0 | 0 | 0 | 0 | 0 | 0 | 0 | 0 | 0 | 0 | 0 | 0 | 0 | 0 | 0 |
| | | | | 3-D | **4** | **1** | 0 | 0 | 0 | 0 | 0 | 0 | 0 | 0 | 0 | 0 | 0 | 0 | 0 | 0 | 0 | 0 | 0 |
| | | | | 3-E | **4** | **1** | 0 | 0 | 0 | 0 | 0 | 0 | 0 | 0 | 0 | 0 | 0 | 0 | 0 | 0 | 0 | 0 | 0 |
| | C5 | 50 | 1 | 1-N | **4** | **1** | 0 | 0 | 0 | 0 | 0 | 0 | **1** | 0 | 0 | 0 | 0 | 0 | 0 | 0 | 0 | 0 | 0 |
| | | | | 1-O | **4** | **1** | 0 | 0 | 0 | 0 | 0 | 0 | **1** | 0 | 0 | 0 | 0 | 0 | 0 | 0 | 0 | 0 | 0 |
| | | | 3 | 3-F | **4** | **1** | 0 | 0 | 0 | 0 | 0 | 0 | 0 | 0 | 0 | 0 | 0 | 0 | 0 | 0 | 0 | 0 | 0 |
| | | | | 3-G | **4** | **1** | 0 | 0 | 0 | 0 | 0 | 0 | 0 | 0 | 0 | 0 | 0 | 0 | 0 | 0 | 0 | 0 | 0 |
| | | | | 3-H | **4** | **2** | 0 | 0 | 0 | 0 | 0 | 0 | 0 | 0 | 0 | 0 | 0 | 0 | 0 | 0 | 0 | 0 | 0 |
| | | | | 3-I | **4** | **3** | 0 | 0 | 0 | 0 | 0 | 0 | 0 | 0 | 0 | 0 | 0 | 0 | 0 | 0 | 0 | 0 | 0 |
| | | | | 3-J | **4** | **3** | 0 | 0 | 0 | 0 | 0 | 0 | **1** | 0 | 0 | 0 | 0 | 0 | 0 | 0 | 0 | 0 | 0 |
| | | | | 3-K | **4** | **2** | 0 | 0 | 0 | 0 | 0 | 0 | **1** | 0 | 0 | 0 | 0 | 0 | 0 | 0 | 0 | 0 | 0 |
| | | | | 3-L | **4** | **2** | 0 | 0 | 0 | 0 | 0 | 0 | **1** | 0 | 0 | 0 | 0 | 0 | 0 | 0 | 0 | 0 | 0 |
| | | | | 3-M | **4** | **3** | 0 | 0 | 0 | 0 | 0 | 0 | **1** | 0 | 0 | 0 | 0 | 0 | 0 | 0 | 0 | 0 | 0 |
| | C6 | 200 | 1 | 1-P | **4** | **3** | 0 | 0 | 0 | 0 | 0 | 0 | 0 | 0 | 0 | 0 | 0 | 0 | 0 | 0 | 0 | 0 | 0 |
| | | | 3 | 3-N | **4** | **3** | 0 | 0 | 0 | 0 | 0 | 0 | **1** | 0 | 0 | 0 | 0 | 0 | 0 | 0 | 0 | 0 | 0 |
| | | | | 3-O | **4** | **4** | 0 | 0 | 0 | 0 | 0 | 0 | **1** | 0 | 0 | 0 | 0 | 0 | 0 | 0 | 0 | 0 | 0 |
| | | | | 3-P | **4** | **4** | 0 | 0 | 0 | 0 | 0 | 0 | **2** | 0 | 0 | 0 | 0 | 0 | 0 | 0 | 0 | 0 | 0 |
